# A Wor1-Like Transcription Factor Is Essential for Virulence of *Cryptococcus neoformans*

**DOI:** 10.3389/fcimb.2018.00369

**Published:** 2018-11-13

**Authors:** Hugo Costa Paes, Lorena da Silveira Derengowski, Luisa Defranco Ferreira Peconick, Patrícia Albuquerque, Georgios Joannis Pappas, André Moraes Nicola, Fabiana Brandão Alves Silva, Marcelo Afonso Vallim, J. Andrew Alspaugh, Maria Sueli Soares Felipe, Larissa Fernandes

**Affiliations:** ^1^Clinical Medicine Division, University of Brasília Medical School, Brasília, Brazil; ^2^Military College, Brasília, Brazil; ^3^Ceilândia College, University of Brasília, Brasília, Brazil; ^4^Department of Cell Biology, Institute of Biological Sciences, University of Brasília, Brasília, Brazil; ^5^Pathology Division, University of Brasília Medical School, Brasília, Brazil; ^6^Department of Biomedicine, Paulista University, Brasília, Brazil; ^7^Cellular and Molecular Biology Division, Biological Sciences Department, São Paulo Federal University, São Paulo, Brazil; ^8^Department of Medicine, School of Medicine, Duke University, Durham, NC, United States; ^9^Graduate School in Genomic Sciences, Catholic University of Brasília, Brasília, Brazil

**Keywords:** *Cryptococcus neoformans*, capsule, cytokinesis, virulence, transcription factor

## Abstract

Gti1/Pac2 transcription factors occur exclusively in fungi and their roles vary according to species, including regulating morphological transition and virulence, mating and secondary metabolism. Many of these functions are important for fungal pathogenesis. We therefore hypothesized that one of the two proteins of this family in *Cryptococcus neoformans*, a major pathogen of humans, would also control virulence-associated cellular processes. Elimination of this protein in *C. neoformans* results in reduced polysaccharide capsule expression and defective cytokinesis and growth at 37°C. The mutant loses virulence in a mouse model of cryptococcal infection and retains only partial virulence in the *Galleria mellonella* alternative model at 30°C. We performed RNA-Seq experiments on the mutant and found abolished transcription of genes that, in combination, are known to account for all the observed phenotypes. The protein has been named Required for cytokinesis and virulence 1 (Rcv1).

## Introduction

Human pathogens of the *Cryptococcus* genus are the causative agents of cryptococcosis, an opportunistic fungal illness affecting immunocompromised patients. Most patients with this infection have significant defects in CD4-mediated immunity, such as those who are co-infected with HIV (reviewed in McDermott and Klein, 2018). The most common agents, belonging to the *C. neoformans* complex, are cosmopolitan yeasts found in nature in association with plants and bird droppings. Primary infections in apparently immunocompetent patients are also reported, mainly for species of the *C. gattii* (Byrnes et al., 2011) complex. In more resource-rich countries, other immunocompromised populations are also at risk for cryptococcosis, such as those receiving immunosuppression for organ transplantation, autoimmune diseases, and cancer therapy (Pyrgos et al., 2013). As with other systemic mycoses, treatment is often prolonged, expensive and marginally effective: in areas lacking adequate access to health care, as many as 80% of all cryptococcosis cases have lethal outcomes, depending on the series reported (Rajasingham et al., 2017). *Cryptococcus* displays many cellular attributes associated with virulence, including its ability to grow at 37°C, the production of a surface polysaccharide capsule, melanisation of the yeast cell, and secretion of enzymes that facilitate tissue invasion and immune system evasion (Srikanta et al., 2014).

To better understand how fungal pathogens cause disease, it is essential to elucidate the detailed mechanisms whereby these microorganisms adapt to the host by changing their morphology and by expressing virulence determinants. Transcription factors (TFs) are at the final level of signaling pathways that convey external information to the genome, and are directly responsible for modulating gene expression in response to environmental cues. Thus, correlating TF activity to specific microbial phenotypes is one way of classifying regulatory units and signaling pathways required for survival in the host.

In *Cryptococcus*, much progress has been made recently to characterize the role of TF families in virulence and adaptability to the host (Jung et al., 2015), and some TFs have been implicated in virulence by non-standard mechanisms (Liu et al., 2008). However, at least one important family has not been investigated in detail in this fungus so far: the Pac2/Gti1 family of cAMP-independent TFs.

Pac2 and Gti1 were the earliest defined members of this family of transcription factors. Discovered about 20 years ago in *Schizosaccharomyces pombe*, Pac2 was first implicated in sexual development (Kunitomo et al., 1995), and Gti1 plays a major role in gluconate transport (Caspari, 1997). These proteins are distinguished by a unique, N-terminal DNA-binding domain composed of two globular sub-domains that are a signature of the family (Lohse et al., 2010). Their C-terminal regions are highly variable and show little conservation from one species to another (Lohse et al., 2010), which possibly reflects the fact that these proteins show a high degree of functional specialization across fungal species.

Pac2/Gti1 proteins are found exclusively in fungi, and they have been most extensively studied in ascomycetes. The Wor1 homolog protein in *C. albicans* controls the white-opaque phenotypic switch, a process that is essential for its parasexual cycle as well as determining the tissue tropism of this microorganism (Huang et al., 2006; Srikantha et al., 2006; Zordan et al., 2006). In the dimorphic fungus *Histoplasma capsulatum*, the Ryp1 protein controls the transition between hyphal and yeast forms as well as the production of spores (Nguyen and Sil, 2008). In *Saccharomyces cerevisiae*, Mit1 controls pseudohyphal growth (Cain et al., 2012). In plant pathogens *Fusarium oxysporum* and *Botrytis cinerea*, Sge1 and Reg1 are required for parasitic growth and in the case of Reg1, also for conidiogenesis and secondary metabolite production (Michielse et al., 2009, 2011). ChIP studies of Ryp1 and Wor1 have shown that these two proteins have a similar DNA binding sites despite functional divergence (Lohse et al., 2010; Beyhan et al., 2013). Studies in the plant pathogen TFs have confirmed nuclear localization of these proteins and the importance of a specific threonine phosphorylation site (Michielse et al., 2009). In almost every fungus studied so far, Pac2/Gti1 proteins have occurred in pairs, each one controlling different processes with varying degrees of overlap (Michielse et al., 2009).

In contrast with the abundance of information in ascomycetes, only two proteins of this family have been studied in basidiomycetes. The *Ustilago maydis* Pac2 homolog controls mating and filamentation in this pathogen of maize (Elías-Villalobos et al., 2011). The *C. neoformans* Pac2 homolog Liv3 was found in a screen for genes required for lung infectivity (Liu et al., 2008), and it was later implicated in quorum-sensing (Homer et al., 2016). We identified the Gti1/Wor1/Ryp1 homolog in *C. neoformans*. The protein was found to control several aspects of fungal virulence and adaptation to the host.

## Results

### General features of the *C. neoformans* Rcv1 protein and its ORF

Using the Ryp1 protein from *H. capsulatum* as query, we performed a BLASTP search against the *C. neoformans* genome (Strain H99–Broad Institute H99 genome database at http://www.fungidb.org). The closest homolog to Ryp1 was an unannotated ORF, CNAG_01983, encoding a predicted protein with 626 amino acids that is a member of the Gti1/Pac2 family (score, 179; *E*-value, 4 × 10^−50^). The overall similarity by the ClustalW alignment tool was of 24.7%. The same search yielded Liv3 (CNAG_05835) as the second closest homolog, and the only other predicted protein in *C. neoformans* with homology to Ryp1. In a report on the homologs from *F. oxysporum* which built a cladogram including both ORFs from *C. neoformans* (Michielse et al., 2009), CNAG_01983 was found to belong to a cluster of proteins related to *S. pombe* Gti1, whereas Liv3 belongs to the Pac2 branch. CNAG_01983 was named Required for cytokinesis and virulence 1 (Rcv1) to reflect the observed phenotypes (see below). Rcv1 and Ryp1 are reciprocal best hits by BLASTP.

**Figure 1:**
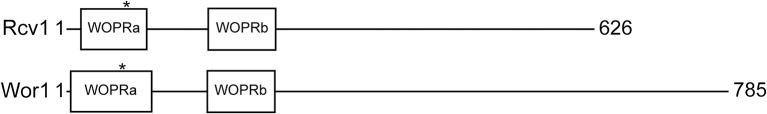
Schematic representation of Rcv1 (top) and Wor1 (bottom) The proteins and their features are drawn to scale. Wor1 is drawn from data of Lohse et al. (2010). The asterisks mark the position of the conserved threonine that is believed to be phosphorylated.

The Rcv1 protein was analyzed by the ELM server domain prediction tool (Puntervoll et al., 2003) to characterize the globular subdomains of the DNA-binding domain (DBD) compared to the related *C. albicans* protein Wor1 (Lohse et al., 2010). The analysis yielded the same result as for Wor1: Rcv1 also has an N-terminal DBD comprised of two globular subdomains (WOPRa on residues 17–95; WOPRb, 168–248), separated by a flexible region (Figure 1). Alignment with other proteins allowed us to identify another feature of proteins from this family that is conserved in Rcv1: the phosphorylation site containing a threonine (Thr^71^) is present in WOPRa (Michielse et al., 2009). The Blast2Seq tool of NCBI, using only the DBD region of Ryp1 (residues 9–227) as query, yields identity of 43% against the 11–248 region of Rcv1.

TFs of the Gti1/Pac2 family are generally implicated in control of fungal morphology changes in response to the environment. The precise nature of these changes varies with each species, indicating a high degree of specialization of these factors. There is strong structural evidence in support of this specialization, in that the only conserved region of these transcription factors is the DNA-binding domain at the N-terminus (Lohse et al., 2010). The C-terminal portion of the protein shows no conservation, which suggests that this region is responsible for the specific interactions of each TF and their species-specific roles.

### Targeted deletion of the *C. neoformans* RCV1 gene and *in vitro* phenotypic analysis

We mutated the *RCV1* gene in the H99 strain background, and the mutant was validated by Southern blotting (Figure S1). To ensure that any potential mutant phenotypes were associated with this mutation, we also created an *rcv1*+*RCV1* reconstituted strain by restoring the original ORF into the mutant strain. The reconstituted strain was confirmed by quantitative RT-PCR to document restored transcript, resulting in full functional restoration of the wild-type phenotype.

The mutant strain, when grown on YPD agar at 30°C, shows no distinct colony or cell morphology. Similarly, most of the commonly tested *in vitro* phenotypes are unchanged for the *rcv1*Δ mutant relative to the wild-type strain (Figure S2). Its susceptibility to various stressing agents, as well as its ability to secrete virulence factors, such as melanin, urease and phospholipase, are not different from the wild type, except for a slight growth deficit in Congo Red at 37°C, which is probably explained by a growth deficit at that temperature (see below).

**Figure 2:**
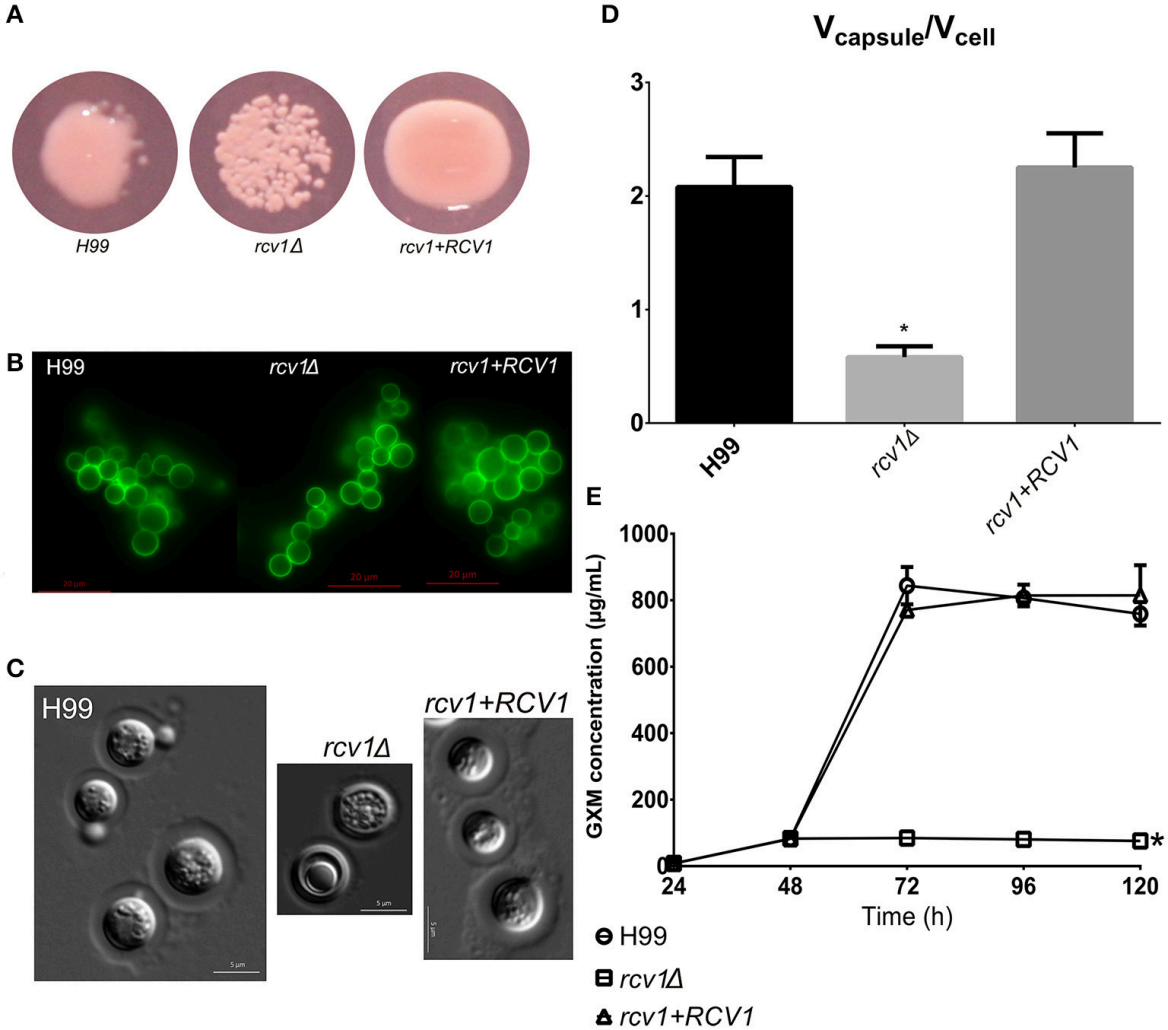
The *rcv1*Δ strain produces capsule, but fails at induction. **(A)** The mutant colonies appear dry on capsule-inducing solid medium at 37°C. DMEM-MOPS, 72 h. **(B)** GXM detection by the 2D10 mAb. The clumping is an artifact caused by the antibody, an IgM. Fluorescence. Sabouraud/MOPS, 24 h, 37°C. **(C)** Percoll^®;^ observation of the capsule. The mutant has a thin, but visible capsule. DMEM-MOPS, 120 h. **(D)** Comparison of the mean ratios of capsule and cell volumes for cells of each strain (50 per group). DMEM/MOPS, 5 days, 37°C. *p < 0.0001, Tukey's post-test, one-way ANOVA. Bars, CI95%. **(E)** GXM secretion pattern, as determined by ELISA, of the mutant strain on minimal medium relative to H99 and the reconstituted strains. This plot is representative of two experiments, each one done in duplicate. Means with SDs. *p < 0.05, Dunnett's multiple comparisons test, two-way ANOVA.

However, the mutant shows a markedly reduced capsule. This phenotype was evident not only in liquid medium but also manifesting as a dry colony on DMEM/MOPS agar, which we found to indicate low capsularity (Figure 2A). Using the 2D10 monoclonal antibody that detects the main capsule component glucuronoxylomannan (GXM), we determined that GXM is present in all mutant and wild type cells (Figure 2B). Moreover, the mutant cells had a thin, but visible capsule on Percoll^®;^ (Figure 2C; Paes et al., 2018). However, the marked reduction in the capsule:cell volume ratio between wild-type and mutant strains (Figure 2D) suggests that while the *rcv1*Δ strain is capable of synthesizing a basal level of capsular polysaccharide, it fails at capsule induction.

Capsule synthesis has been correlated with nonconventional vesicle secretion pathways in *C. neoformans* (Rodrigues et al., 2007). However, the same pathways have also been shown to be essential for melanin secretion (Eisenman et al., 2009), and correlated with urease secretion (Rodrigues et al., 2008), two phenotypes that are not altered in the *rcv1*Δ mutant. The mutant strain also secretes phospholipase normally. There are at least two possible explanations for this observation: (a) the *rcv1*Δ mutant has normal vesicle secretion but has a defect in incorporation of components to the outer layers of the capsule; or (b) there are subsets of vesicles with different functions and cargo, and the *rcv1*Δ mutant has a specific defect in the secretion of GXM. To test for it, we performed a secreted GXM ELISA comparing the *rcv1*Δ and H99 strains. As shown in Figure 2E, the mutant strain has a severe GXM secretion defect: capsule polysaccharide accumulates in the medium in the first 48 h, but not afterwards, whereas in the wild-type strain the GXM concentration rises sharply after that point. This also indicates *C. neoformans* secretes GXM in a bimodal way. This observation is in agreement with previous work that has shown that there are several extracellular vesicle morphologies, perhaps delivering different cargo (Rodrigues et al., 2008). A third explanation, which cannot be ruled out by these results, is that the mutant has a regulation defect that abrogates the induction of GXM synthesis after 48 h.

### The *rcv1Δ* mutant has growth defects at 37°C

**Figure 3:**
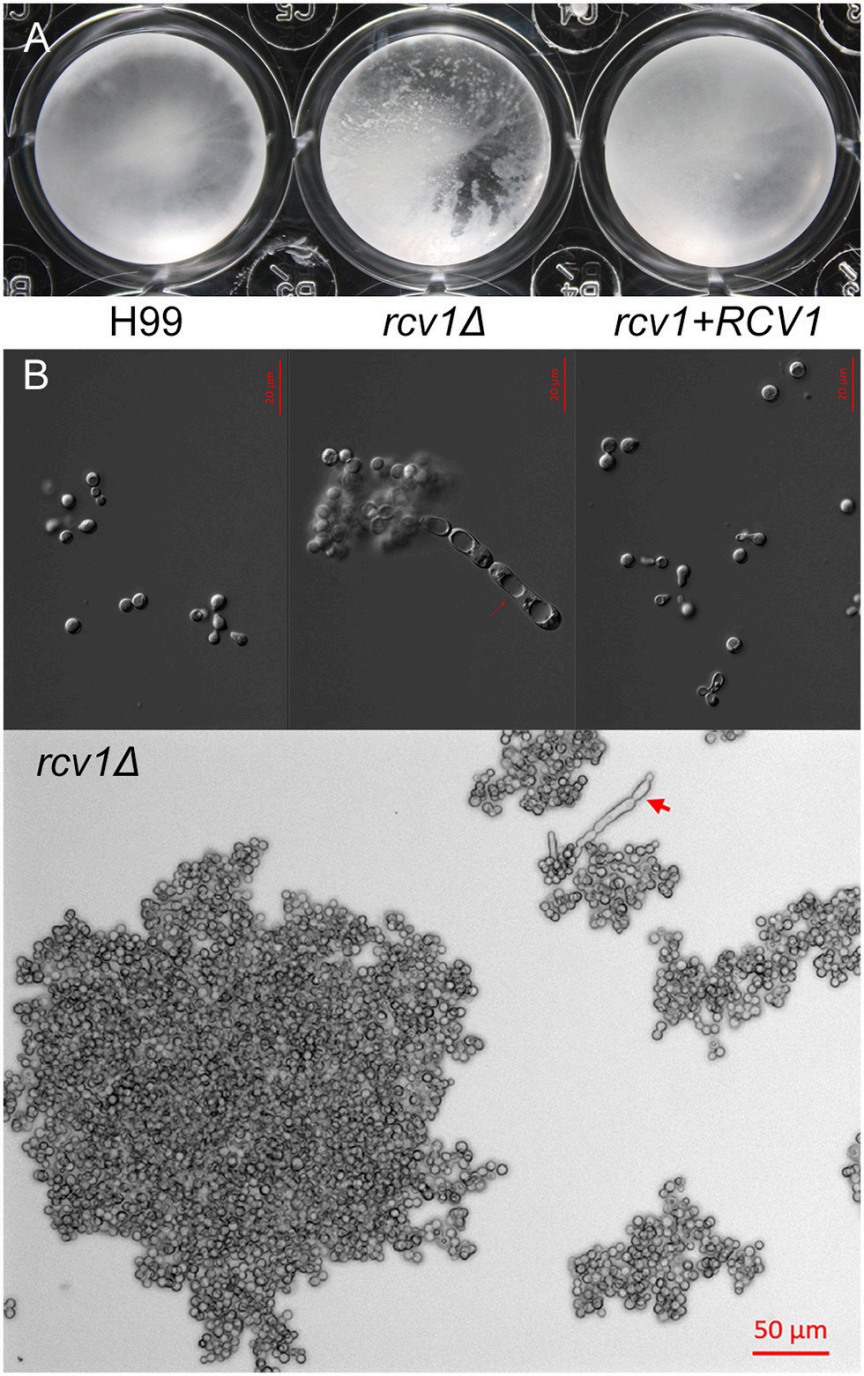
The *rcv1*Δ strain shows heavy clumping under certain conditions. **(A)** Clumping of cells becomes macroscopically apparent for the mutant strain after 9 days at 37°C on Sab/MOPS. **(B)** Upper panel: DIC micrographs of yeast cells from the three strains after 7 days on CO_2_-independent medium at 37°C. Notice the elongated cells with prominent vacuoles on the mutant. Lower panel: large clump of *rcv1*Δ cells on same conditions. Arrows, pseudohypha-like structures.

Although colonies of the *rcv1*Δ strain did not show reduced size on solid media incubated at 37°C (Congo Red and DMEM/MOPS; Figure S2), we often observed that cell cultures in liquid media at this temperature yielded lower cell densities relative to the wild-type strains. Thus, we decided to assess the growth profile of the mutant in liquid media. Since *C. neoformans* only grows on DMEM/MOPS agar in the presence of 5% CO_2_, we replaced it with CO_2_-independent medium (CIM), which also induces capsule in this fungus. The growth curves in Figure 3 show that, during the exponential phase, the mutant strain has a longer doubling time at 37°C in both media, and at 30°C in CIM, relative to the wild-type and reconstituted strains. Furthermore, in the same conditions the *rcv1*Δ strain reached stationary phase at a lower density than H99 and *rcv1*Δ + *RCV1*.

**Figure 4:**
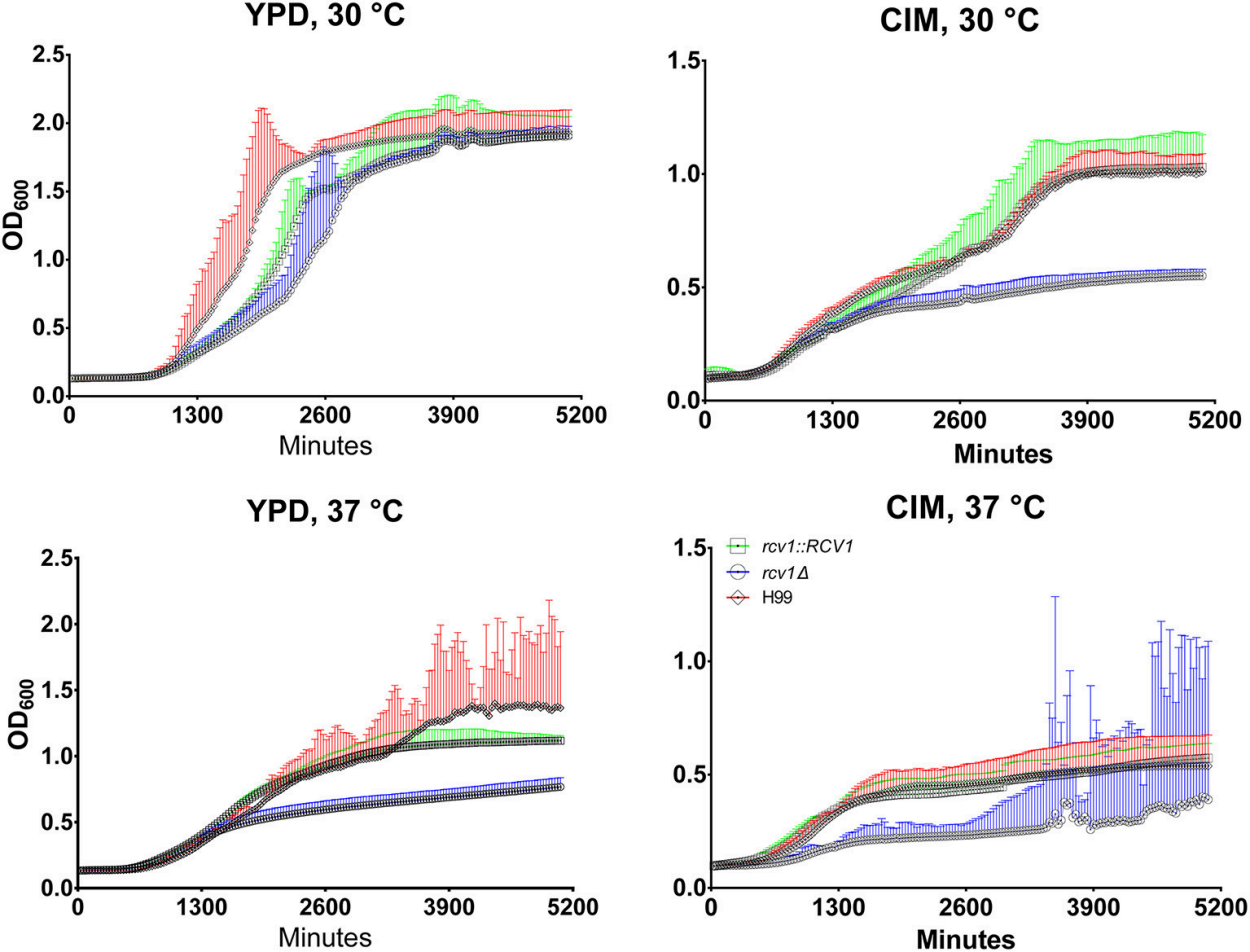
At 37°C or on cell culture medium, the *rcv1*Δ mutant shows retarded growth and reaches stationary phase at lower densities. *C. neoformans* yeast cells were inoculated in triplicate at a density of 10^4^ cells/mL and incubated at the indicated media and temperatures under orbital shaking and cultures had their OD_600_ measured every 30 min. The high dispersion in measurements observed at later time points for the mutant strain on CIM at 37°C was caused by heavy clumping of cells. The experiment is representative of two and each sample was processed in triplicate. Error bars are 95% confidence intervals (CI95%) and the same color of the curves (red, H99; blue, *rcv1*Δ; green, *rcv1* + *RCV1*).

Interestingly, the *rcv1*Δ strain grown at 37°C in CIM displays extensive cell clumping that disturbs optical density measurements. The mutant strain clumps after prolonged incubation not only in CIM, but also in another capsule-inducing medium, Sabouraud/MOPS (Zaragoza and Casadevall, 2004; Figures 4, S3). Longer incubation periods result in clumps containing hundreds of cells, as well as the appearance of cells with aberrant morphology, including elongated chains of cells. While temperature accentuates the phenotype, and so do capsule-inducing media, Figure S3 shows that even in YPD at 30°C there is modest clumping of cells.

To distinguish between simple electrostatic interactions and cytokinesis defects that preclude separation of daughter cells from their progenitors, we performed time-lapse microscopy (see Videos S1–S3) of cell division in CIM at 37°C. The images demonstrated that *rcv1*Δ cells grow into large clumps with sporadic release of grouped cells, whereas the wild-type yeast can be seen fully to detach from their mother cells during division, and solitary cells often drift away from the group of dividing cells. Furthermore, sonication of clumps for 20 or 60 s did not detach the cells, even when the clumps began to be destroyed by the procedure (Figure S4). The *rcv1*Δ strain, therefore, has a cell separation defect that worsens at host physiological temperatures and results in marked cell clumping.

The mutant strain was co-incubated with J774A.1 macrophages to assess rates of phagocytosis and fungal survival in this system. After 24 h, the macrophages were lysed, and the number of colony-forming units (CFUs) was estimated by quantitative culture. As shown in Figure S5, the *rcv1*Δ strain yields lower CFU numbers when recovered from within macrophages relative to the parental and reconstituted strains. The phagocytic index at 2 h of co-incubation was about 50% for all strains, which indicates that the survival differences observed were not due to reduced uptake of the mutant strain. However, the precise cause of this observation cannot be determined with certainty: it can be due to increased killing by the macrophage, a lower division rate at 37°C, an artificial decrease in CFU caused by clumping of mutant cells as described above, or more likely by a combination of these factors.

To assess whether clumping and temperature were the sole factors that made the mutant susceptible to killing by phagocytes, the same assay was performed at 30°C with *Acanthamoeba castellanii*, a well-established model for analysis of cryptococcal virulence *in vitro* (Steenbergen et al., 2001). We found that the CFU numbers were reduced by more than one order of magnitude for the mutant strain (Figure S5), suggesting that at least for this phagocyte, other factors than high temperature growth and cytokinesis defects are at play.

### The *rcv1Δ* strain shows decreased virulence *in vivo*

**Figure 5:**
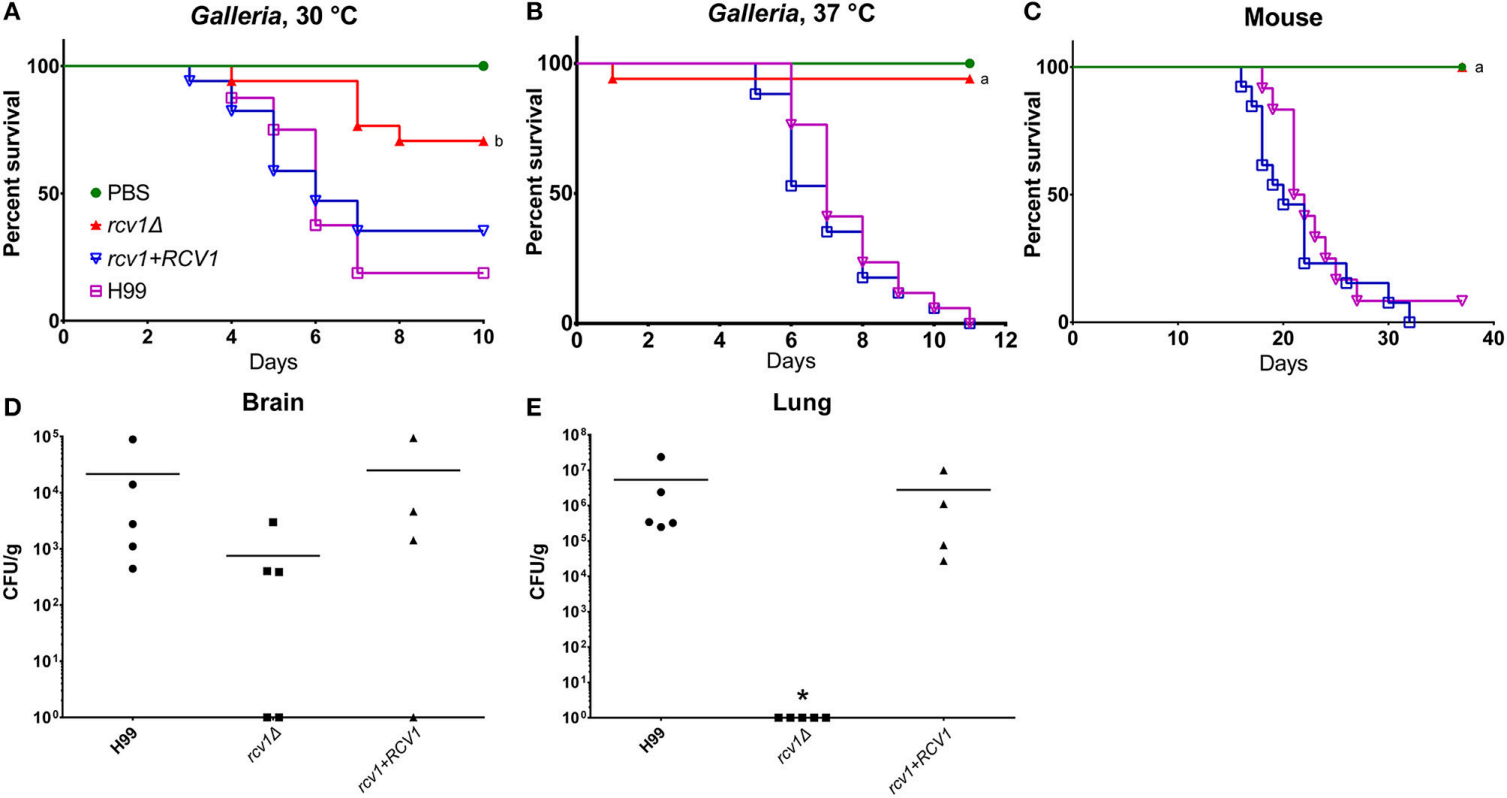
The *rcv1*Δ strain is hypo- or avirulent *in vivo*. **(A–C)** Survival curves in caterpillar and murine models. *G. mellonella* were infected with 10^4^ yeast cells per individual, whereas BALB/C mice were infected with 2 × 10^5^ cells each. The “a” indicates statistical difference relative to H99 and *rcv1*+*RCV1*, and “b” indicates statistical difference relative to all the other groups (log-rank test, *p* < 0.001). **(D,E)** Fungal burdens in brain and lung from mice at day 12 of infection. Groups were compared using the Kruskal-Wallis test. The asterisk indicates significance (*p* < 0.05) relative to the other two groups. Bars, means.

The altered survival of the *rcv1*Δ strain in macrophages at 37°C suggested a defect in virulence. To confirm this with *in vivo* models of host-microbe interaction, we inoculated *Galleria mellonella* larvae with the wild-type, *rcv1*Δ mutant, and *rcv1* + *RCV1* reconstituted strains. In this assay, the *rcv1*Δ strain was completely avirulent at 37°C and hypovirulent at 30°C relative to wild-type, indicating that virulence loss in this mutant is only partially explained by a defect in adaptation to the temperatures of the mammalian host (Figures 5A–C). The *rcv1*Δ mutant was also tested for virulence in a murine intratracheal model of cryptococcal infection. Mice infected with the wild-type or reconstituted strains have similar survival, with nearly all mice succumbing to the infection by 30 days after inoculation. In contrast, mice infected with the *rcv1*Δ mutant survived the entire 35 days of the observed infection period (*p* < 0.001; Figures 5A–C).

### The *rcv1Δ* strain displays asymptomatic persistence in the brain

Figures 5D,E shows the fungal burdens of lung and brain of mice infected with the mutant and wild-type strains for 12 days. At that point, the *rcv1*Δ strain had been completely cleared from the lungs in all individuals but, surprisingly, it was still recovered from the brain, even though the animals showed no neurological signs or symptoms. The burden seemed reduced relative to H99 and *rcv1*Δ + *RCV1*, even though due to wide dispersion, the difference was not statistically significant. However, we were also able to retrieve yeast cells from the brain of mice infected for 100 days at a mean density of 3.5 × 10^5^ CFU/g and found they had been cleared only after 6 months. We believe this is the longest asymptomatic persistence yet reported for a *C. neoformans* mutant in the CNS of its host.

The absence of noticeable signs of illness even while there are yeast cells in the brain may be due to several factors, including the heat sensitivity for cell division in the *rcv1*Δ background and the inability to induce thickening of the capsule in response to environmental cues. The production of capsule and the doubling rate of the cell have been correlated with the onset of meningoencephalitis (Pool et al., 2013), and the mutant is defective for both.

**Figure 6:**
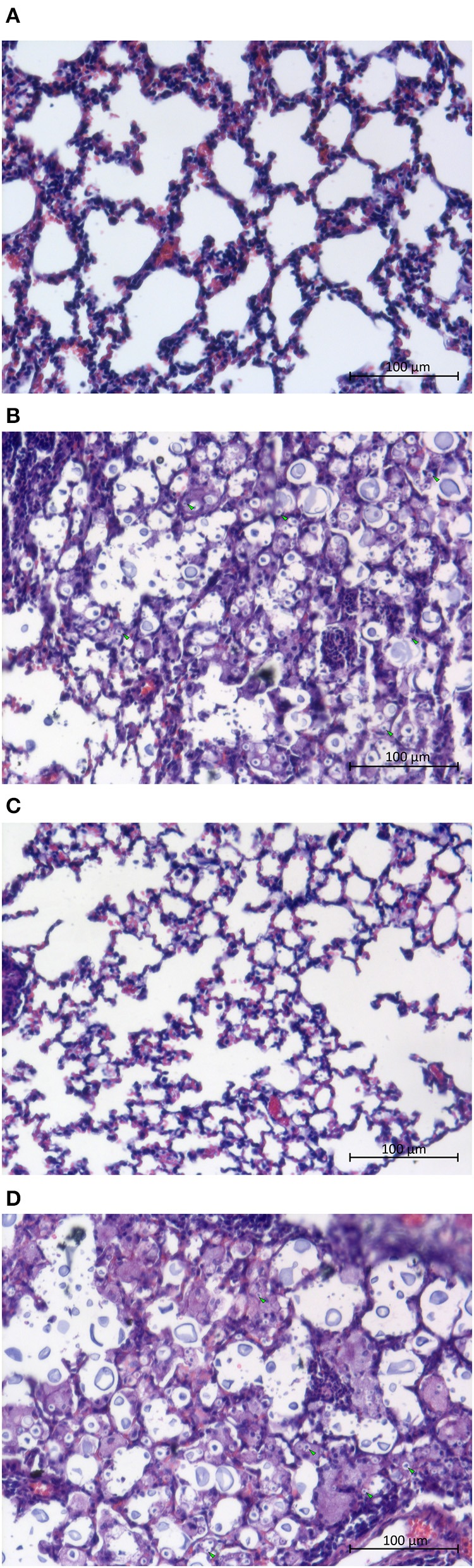
The *rcv1*Δ strain does not cause damage to the lungs. Mice were infected as in Figure 5 (4–5 individuals per group) and lungs were harvested at day seven. **(A)** Normal lung. **(B)** H99 infection. **(C)** *rcv1*Δ infection. **(D)** *rcv1*+*RCV1* infection. HE staining, bright field. Green arrowheads, five randomly selected yeast cells per field in **(A,C)**, for illustration.

Despite the fact that *rcv1*Δ yeast cells reach the CNS, the lung parenchyma appears intact on HE staining (Figure 6), whereas the wild-type and *rcv1* + *RCV1* strains cause widespread inflammation with monocytic and lymphocytic infiltration and disruption of the alveolar architecture that are hallmarks of pulmonary cryptococcosis. This suggests that clearance of the mutant cells from the lung is fast, and that the fungus reaches the brain before there is significant colonization of the lungs.

### The Rcv1 protein localizes to the nucleus

**Figure 7:**
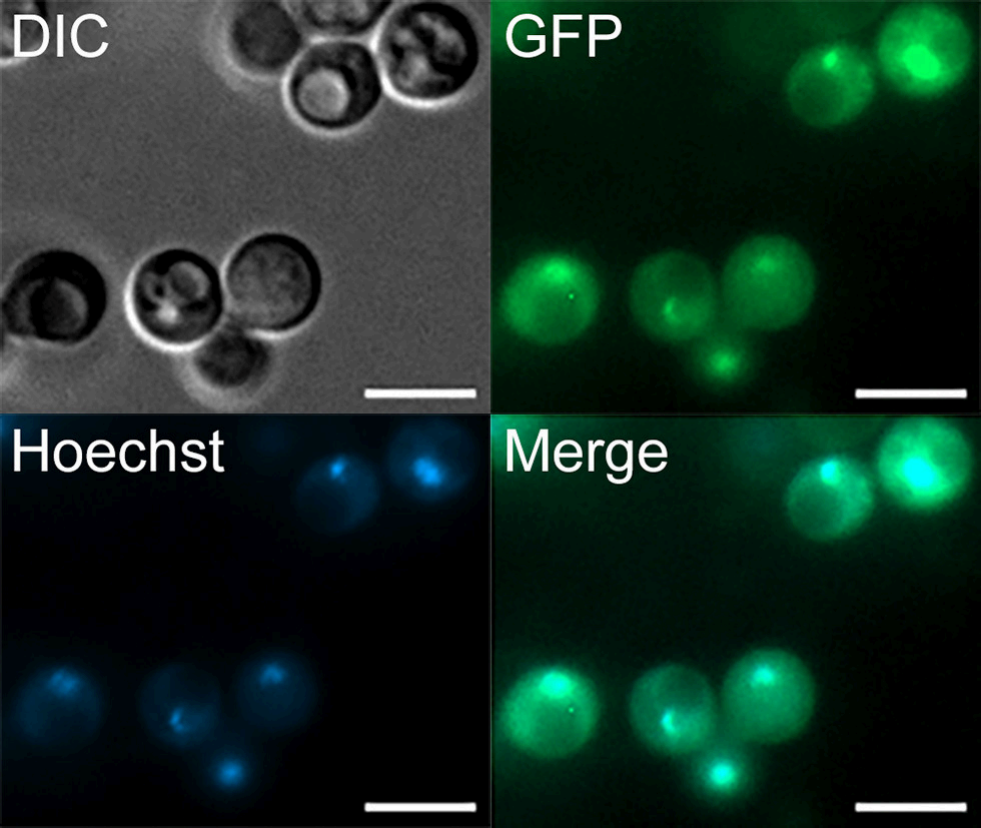
The Rcv1-GFP chimera localizes to the nucleus. The *RCV1-GFP* strain was grown for 16 h on YPD at 30°C before cells from the culture were applied to a microscope slide. Rcv1-GFP cells were stained with Hoechst^®;^ 33342, showing that the nuclear stain co-localizes with the GFP signal. Bar, 5 μm. DIC, differential interference contrast microscopy. See Methods for details.

We have shown by sequence analysis that Rcv1 has features characteristic of a transcription factor family protein. To test whether the protein localizes to the nucleus as expected of a TF, we transformed the *rcv1*Δ strain with an *RCV1-GFP* construct created from the pCN50 plasmid (O'Meara et al., 2010), which drives transcription of the construct from the native histone H3 promoter. The *RCV1* gene was inserted into the 3′ region of the *GFP* gene, resulting in a C-terminal fusion. The chimaeric protein did localize to the nucleus, as shown by the identical topology of the GFP signal and the DNA staining with Hoechst 33342 (Figure 7). That topology was observed during vegetative growth on YPD at 30°C, so Rcv1 seems to be constitutively nuclear for the most part. However, the Rcv1-GFP fusion protein does not reconstitute the wild-type phenotypes.

### Absence of the Rcv1 protein results in changes in the transcription profile

Because all the *in vitro* phenotypes of *rcv1*Δ–absence of capsule induction, clumping and growth retardation–can be observed in CIM at 37°C, we chose this condition to profile the transcriptional programme of the *rcv1*Δ strain by RNA-Seq at 4 h of incubation.

As shown in Table 1, transcripts of 67 annotated ORFs were found to be differentially regulated above the 2-fold cut-off in the mutant relative to the wild-type strain, and 35 of those had functional annotations. The gene set was divided between up-regulated (25) and down-regulated (42) ORFs in the mutant, though the latter group predominated.

**Table 1:** Differential^#^ transcripts in the *rcv1*Δ mutant after 4 h in CIM at 37°C, relative to H99.

**ID**	**Name**	**Chromosome**	**Start**	**End**	**Strand**	**GO**	**Fold-change^a^**	***q*-value^b^**
**UP-REGULATED**								
CNAG_00165	Methylthioadenosine phosphorylase	Chr_01	450085	451511	–	GO:0044281, GO:0016757, GO:0034641, GO:0009058, GO:0005622	1.31976	0.0017488
CNAG_00984	Glucose and ribitol dehydrogenase	Chr_05	1505336	1507350	+	GO:0016491, GO:0005622	1.13924	0.0017488
CNAG_02189	Alpha-amylase	Chr_06	950723	953183	–	GO:0005623, GO:0005975, GO:0009056, GO:0043167, GO:0016798	1.37356	0.0017488
CNAG_05803	Exo-beta-1, 3-glucanase	Chr_07	915846	919310	–	GO:0005623, GO:0005975, GO:0007155, GO:0071554, GO:0016798	1.24897	0.0017488
CNAG_03572	OPS1 opsin 1	Chr_08	1304364	1306280	+	GO:0022857, GO:0005575	1.09304	0.0017488
CNAG_04735	MEP1 extracellular elastinolytic metalloproteinase	Chr_10	544141	547378	+	GO:0005615, GO:0043167, GO:0008233	2.14455	0.0017488
CNAG_01612	CAMK/CAMKL protein kinase	Chr_11	417056	420512	+	GO:0043167, GO:0006464, GO:0016301, GO:0005622	3.69986	0.0017488
CNAG_01947	2, 4-dienoyl-CoA reductase	Chr_11	1338854	1340282	+	GO:0016491	1.4266	0.0017488
CNAG_06323	L-fucose permease	Chr_13	188665	191970	+	GO:0055085, GO:0005575	1.15098	0.0017488
CNAG_00505	Hypothetical protein	Chr_01	1289931	1292528	+	GO:0005634, GO:0001071, GO:0043167, GO:0034641, GO:0009058	1.20452	0.0017488
CNAG_06800	Hypothetical protein	Chr_02	5236	6136	+		1.14702	0.0017488
CNAG_03058	Hypothetical protein	Chr_03	98696	99468	+		1.09043	0.0017488
CNAG_02684	Hypothetical protein	Chr_03	1023010	1028604	+		1.51902	0.0017488
CNAG_06926	Hypothetical protein	Chr_03	1535763	1539503	–	GO:0055085, GO:0005575	1.39577	0.0017488
CNAG_01384	Hypothetical protein	Chr_05	436320	439953	+	GO:0055085, GO:0048856, GO:0005575	1.07407	0.0017488
CNAG_02558	Hypothetical protein	Chr_06	2123	3095	+		1.58286	0.0017488
CNAG_02070	Hypothetical protein	Chr_06	1279200	1281525	+		1.1974	0.0017488
CNAG_03454	Hypothetical protein	Chr_08	991849	993501	–		1.77805	0.0017488
CNAG_03495	Hypothetical protein	Chr_08	1106108	1107414	+	GO:0003674	1.49564	0.0133242
CNAG_04903	Hypothetical protein	Chr_10	71513	73231	+		1.15643	0.0017488
CNAG_04737	Hypothetical protein	Chr_10	536862	541075	–		1.14503	0.0017488
CNAG_04736	Hypothetical protein	Chr_10	541811	543861	–		1.19209	0.0017488
CNAG_01527	Hypothetical protein	Chr_11	187192	188874	–		1.82243	0.0017488
CNAG_06267	Hypothetical protein	Chr_13	34262	36703	–	GO:0048856	1.28114	0.0017488
CNAG_05641	Hypothetical protein	Chr_14	853580	854667	–		1.34175	0.0017488
**DOWN-REGULATED**								
CNAG_00141	Monooxygenase	Chr_01	380990	382651	+	GO:0019748, GO:0016491, GO:0043167, GO:0009058	−1.69077	0.0017488
CNAG_03911	Hydrolase	Chr_02	1163627	1168132	–		−1.18501	0.0017488
**CNAG_06815**	**FAO1 fatty alcohol oxidase (putative)**	**Chr_05**	**183563**	**185651**	+	**GO:0016491, GO:0043167**	**#DIV/0!**	**0.0017488**
**CNAG_06812**	**SPO14**α **phospholipase D1**	**Chr_05**	**193323**	**200778**	**–**	**GO:0007165, GO:0009056, GO:0043167, GO:0008289, GO:0009058, GO:0043226, GO:0005886, GO:0006950, GO:0040007, GO:0002376, GO:0048870, GO:0016192, GO:0005737, GO:0006629**	−**4.37317**	**0.0017488**
**CNAG_06811**	**RPL22**α **large subunit ribosomal protein L22e**	**Chr_05**	**200908**	**202000**	+	**GO:0003735, GO:0048856, GO:0005829, GO:0042254, GO:0005730, GO:0005840, GO:0006412**	−**8.0929**	**0.0017488**
**CNAG_06809**	**IKS protein kinase**	**Chr_05**	**204043**	**207446**	**–**	**GO:0043167, GO:0006464, GO:0016301**	−**7.1915**	**0.0017488**
**CNAG_06806**	**ETF1**α **electron transfer flavoprotein alpha subunit**	**Chr_05**	**212067**	**213908**	+	**GO:0005739, GO:0043167, GO:0048856**	−**7.82241**	**0.0017488**
**CNAG_07407**	**MF**α**3 fungal mating-type pheromone**	**Chr_05**	**222703**	**223313**	+		**#DIV/0!**	**0.0017488**
**CNAG_06971**	**MYO2 myosin class V heavy chain**	**Chr_05**	**223845**	**230309**	+	**GO:0061024, GO:0043167, GO:0048856, GO:0005783, GO:0043234, GO:0005576, GO:0005764, GO:0005777, GO:0005768, GO:0005856, GO:0003723, GO:0016192, GO:0005829, GO:0016023, GO:0019899**	−**7.12373**	**0.0017488**
**CNAG_07408**	**STE/STE20/PAKA protein kinase**	**Chr_05**	**230210**	**233166**	**–**	**GO:0008283, GO:0007010, GO:0006461, GO:0043167, GO:0006464, GO:0006520, GO:0008289, GO:0009058, GO:0008219, GO:0034641, GO:0050877, GO:0061024, GO:0007165, GO:0000902, GO:0007267, GO:0048646, GO:0006810, GO:0005886, GO:0002376, GO:0006950, GO:0007049, GO:0048870, GO:0005634, GO:0005856, GO:0051276, GO:0042592, GO:0005739, GO:0030154, GO:0016023, GO:0016301, GO:0019899**	−**7.49372**	**0.0017488**
**CNAG_06980**	**STE/STE11 protein kinase**	**Chr_05**	**233495**	**238298**	+	**GO:0019748, GO:0007010, GO:0043167, GO:0006464, GO:0048856, GO:0006520, GO:0009058, GO:0004871, GO:0000003, GO:0007049, GO:0006950, GO:0040007, GO:0005635, GO:0051301, GO:0008219, GO:0005737, GO:0016301**	−**7.73226**	**0.0017488**
**CNAG_07411**	**RUM1**α **transcription factor**	**Chr_05**	**256514**	**263235**	+	**GO:0005634, GO:0003677, GO:0016491, GO:0051276, GO:0043167, GO:0008168, GO:0034641, GO:0009058**	−**4.4086**	**0.0017488**
**CNAG_01457**	**BSP1 hypothetical protein**	**Chr_05**	**263566**	**266560**	+	**GO:0004518, GO:0034655, GO:0000228, GO:0043234, GO:0009058, GO:0006259, GO:0006950**	−**5.2879**	**0.0017488**
**CNAG_01455**	**RPL39**α **large subunit ribosomal protein L39**	**Chr_05**	**270517**	**271940**	+	**GO:0061024, GO:0042254, GO:0005840, GO:0044403, GO:0006950, GO:0002376, GO:0006412, GO:0006605, GO:0005615, GO:0003723, GO:0003735, GO:0005739, GO:0034655, GO:0005829**	−**8.38587**	**0.0017488**
**CNAG_01454**	**Transcription factor STE12**α	**Chr_05**	**271943**	**275729**	**–**	**GO:0005634, GO:0003677, GO:0001071, GO:0043167, GO:0034641, GO:0009058, GO:0043234, GO:0000003, GO:0006950, GO:0040007**	−**8.06848**	**0.0017488**
CNAG_01040	Carboxypeptidase D	Chr_05	1366524	1369193	–	GO:0008233, GO:0005622	−1.46687	0.0017488
CNAG_00979	CTR4 solute carrier family 31 (copper transporter) member 1	Chr_05	1517505	1520014	+	GO:0022857, GO:0005575	−2.54327	0.0017488
CNAG_02553	Short-chain dehydrogenase	Chr_06	14225	15571	+	GO:0016491	−1.26659	0.0017488
CNAG_05914	MFS transporter SP family general alpha glucoside:H+ symporter	Chr_07	1203508	1206323	+	GO:0022857, GO:0005886	−1.02211	0.0017488
CNAG_04245	CHI22 endochitinase	Chr_09	393733	396949	+	GO:0005975, GO:0009056, GO:0071554, GO:0016798	−1.31553	0.0017488
CNAG_04869	PNB1 para-nitrobenzyl esterase	Chr_10	154082	158000	–	GO:0008150, GO:0003674	−1.9354	0.0017488
CNAG_04837	MLN1 hypothetical protein	Chr_10	272012	274859	–	GO:0003674	−1.56293	0.0017488
CNAG_01572	Tyrosine phosphatase	Chr_11	317869	321637	+	GO:0005634, GO:0005737, GO:0016791, GO:0006464, GO:0006520, GO:0007049, GO:0000003	−0.796825	0.0017488
CNAG_01605	Rossman fold oxidoreductase	Chr_11	398530	400001	+	GO:0016491	−1.04916	0.0017488
CNAG_01611	LIV8 required for lung infection, unknown activity	Chr_11	412787	415620	+		−1.17352	0.0017488
CNAG_05662	ITR4 sugar transporter	Chr_14	908236	910766	+	GO:0022857, GO:0005575	−1.1498	0.0017488
CNAG_02899	Hypothetical protein	Chr_03	500229	501212	+	GO:0005622	−1.18091	0.0017488
CNAG_05079	Hypothetical protein	Chr_04	381088	381903	–		−1.42738	0.0017488
CNAG_05265	Hypothetical protein	Chr_04	883017	884348	–		−1.23005	0.0250451
CNAG_07826	Hypothetical protein	Chr_04	944969	945496	+		−1.0303	0.0017488
**CNAG_06816**	**Hypothetical protein**	**Chr_05**	**182118**	**185465**	**–**	**GO:0008150, GO:0043167**	−**1.17198**	**0.0239309**
**CNAG_06814**	**Hypothetical protein**	**Chr_05**	**185699**	**188858**	+	**GO:0005634, GO:0003677, GO:0034641, GO:0009058**	−**7.27443**	**0.0017488**
**CNAG_06813**	**Hypothetical protein**	**Chr_05**	**189559**	**192240**	**–**		−**3.61882**	**0.0017488**
**CNAG_06807**	**Hypothetical protein**	**Chr_05**	**210556**	**211579**	**–**		**#DIV/0!**	**0.0017488**
**CNAG_06805**	**Hypothetical protein**	**Chr_05**	**214529**	**215069**	+		−**0.916582**	**0.019596**
**CNAG_06804**	**Hypothetical protein**	**Chr_05**	**215849**	**219593**	**–**	**GO:0003674**	−**8.1898**	**0.0017488**
**CNAG_07015**	**Hypothetical protein**	**Chr_05**	**251934**	**255596**	**–**	**GO:0007165, GO:0003674**	−**1.35923**	**0.0017488**
CNAG_05911	Hypothetical protein	Chr_07	1194043	1195763	+		−1.12321	0.0017488
CNAG_03477	Hypothetical protein	Chr_08	1059455	1061314	–		−1.29087	0.0017488
CNAG_01534	Hypothetical protein	Chr_11	214419	215253	+		−1.08504	0.0017488
CNAG_01538	Hypothetical protein	Chr_11	229248	234150	+		−1.08504	0.0017488
CNAG_01560	Hypothetical protein	Chr_11	279255	281611	–		−1.02382	0.0017488
CNAG_01621	Hypothetical protein	Chr_11	447770	449716	+		−2.1384	0.0017488

# *Statistically validated by the CuffDiff suite, and manually filtered for biological relevance by selecting only transcripts with a linear fold-change greater than two*.

a *Fold-change values are given in the base-2 logarithm. #DIV/0! fields mean no transcript was detected in the mutant*.

b *The q-values were obtained from a p-value set at 0.05. The transcripts shaded in bold are the region of chromosome 5 where the MATα locus is located and its immediate vicinity. The greatest changes in expression were observed in transcripts in this area*.

**Figure 8:**
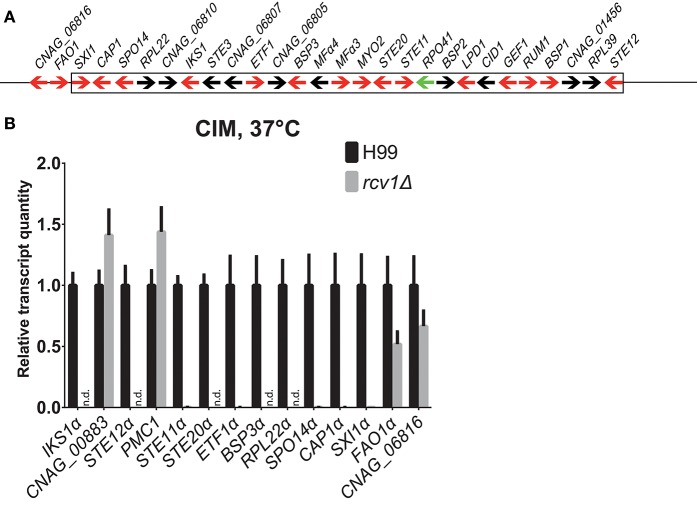
The genes the expression whereof was most strongly affected by Rcv1 deletion are clustered in the *MAT*α locus. **(A)** Map of the *MAT*α locus of H99 (not to scale) based on the latest assembly by the Broad Institute. Arrows indicate annotated ORFs. Those in red are induced in the macrophage according to the work of Heitman and colleagues (Fan et al., 2005) and repressed in the *rcv1*Δ strain in our RNA-Seq data. The only ORF for which data of the two studies are divergent, *RPO41*α, is indicated as a green arrow. The box indicates the borders of the locus and two upstream ORFs that are also repressed in the *rcv1*Δ are also shown. See text for details. **(B)** Quantitative PCR validation of RNA-Seq data for a selection of RNA-Seq differential ORFs. The plots indicate relative transcript quantitation using *ACT1* as the housekeeping transcript and H99, 4 h CIM at 37°C as reference. Bars are SDs. Samples were the same used for the RNA-Seq itself plus two independent RNA extractions for each condition. N.d., not detectable (transcripts were less than 0.01% of wild-type). The *y* axis is linear. See text for details.

One striking observation is that the 15 genes with the largest change in expression in the mutant relative to the wild-type strain have been previously shown to be upregulated upon phagocytosis by macrophages (Fan et al., 2005) and are located in the *C. neoformans MAT* locus (Figure 8A) on chromosome 5. They are repressed, some completely so in the mutant strain. We used real-time PCR to verify the regulation of selected genes on the same and on independently extracted samples. We chose 18 genes to test: the most differential ones, all from the *MAT*α locus, plus four from other regions, two up- and two down-regulated. The two upregulated ones, CNAG_00883 and *PMC1* (CNAG_01232), are not listed in Table 1 because although they met the statistical validation criterion (q-value of 0.0017488 for CNAG_00883 and of 0.0133242 for *PMC1*), they did not reach our biological relevance cut-off (a minimal fold-change of two; in total, ~350 transcripts met the statistical threshold). However, testing them would indicate that even small fluctuations in our RNA-Seq data are reliable, and that was the rationale for selecting them. Of the 18, fourteen (77.8%, Figure 8B) had their expression profile confirmed.

As noted above, this is not the first report of co-regulation of *MAT* locus genes. Fan et al. (2005) have reported similar findings in microarray-based transcriptional studies of *C. neoformans* cells co-incubated with macrophages; in Figure 8A we show the correlation of our findings. Of the 26 ORFs annotated in the current assembly, 16 were upregulated in the macrophage study, and 15 of those are downregulated or completely suppressed in the *rcv1*Δ strain growing on CIM. That includes all the transcripts that crossed the 2-fold cut-off used by the authors of the former study and the ones that were confirmed independently by quantitative PCR. Curiously, the co-regulation module extends a little further upstream of the *MAT*α locus: we found that *FAO1* (a putative fatty-alcohol oxidase just upstream of the locus) and CNAG_06816, a putative protein the coding sequence of which is partially superimposed with that of *FAO1* but on the opposite strand, are also repressed in the *rcv1*Δ mutant.

To better determine how Rcv1 controls that portion of the *MAT*α locus, we performed quantitative PCR comparing the expression of selected genes from that region between mutant and wild-type at vegetative growth (YPD, 30°C, exponential phase), heat challenge (YPD, 37°C) or medium change (CIM, 30°C). The results (Figure S6) show that Rcv1 is necessary to maintain expression of the tested genes at all conditions, and many of the transcripts became undetectable in the *rcv1*Δ mutant (e.g., *STE12*α).

### The loss of Rcv1 does not affect mating negatively

The conditions under which we performed qPCR and RNA-Seq are not permissive for mating in *C. neoformans*. However, seeing as loss of Rcv1 resulted in a shut-down of the *MAT* locus under many *in vitro* conditions, we generated **a** and α *rcv1*Δ mutants from the KN99a and KN99α strains and found that they filament normally when combined on mating-inducing SLAD medium (Figure S7A). These experiments were repeated on Murashige & Skoog agar (MSA) and surprisingly, there appeared to be an increase in filamentation of the mutants during mating (Figure S7B). The *rcv1*Δ mutants in the KN99 background were tested for capsule and cytokinesis, and the phenotypes matched those of the mutant generated from the H99 background (Figure S8). Mating was followed on MS mating medium to the final product of basidiospore production, and the mutants were found to produce viable spores in a qualitatively indistinguishable fashion relative to the wild-type strains (data not shown).

These findings are in sharp contrast with the phenotypes of individual mutants for other genes located within the *MAT*α locus. Whereas, *ste12*α and *ste20*α (in addition to *ste11*α) are mating or haploid fruiting-deficient and do not even show filamentation when combined with their corresponding **a** mutants (Yue et al., 1999; Clarke et al., 2001; Wang et al., 2002), the *rcv1*Δ mutant filaments normally. To account for this observation, we used qPCR to measure transcript levels of *STE12*α, *STE20*α and *RUM1*α in cells grown in SLAD agar for 24 h at room temperature in the presence of the **a** mutant. We chose these genes because in combination, they can account for the *rcv1*Δ phenotype in non-mating conditions (see Discussion). We found that the three genes were transcribed in the mutant at equal or higher levels than in the wild-type strains (Figure S9), indicating that Rcv1 does not regulate *MAT* locus genes under standard laboratory mating conditions, but rather under conditions more likely to mimic the infected host.

## Discussion

In the present work, we report the characterization of a Wor1/Gti1 homolog in *C. neoformans*. In keeping with findings for TFs of the same family in other fungal species, Rcv1 controls aspects of morphology and virulence of *C. neoformans*, and the results obtained show it is necessary for cytokinesis at host physiological temperatures, cell capsule thickening under inducing conditions and colonization of both a mammalian and an invertebrate host. The absence of the *RCV1* gene also causes a severe impairment in GXM production or secretion. We have also obtained evidence that it localizes to the nucleus. While it may be argued that the subcellular localization of a non-functional protein (the Rcv1-GFP chimera) does not necessarily reflect the function of the native counterpart, GFP is not known to localize to the nucleus on its own.

An intriguing observation was that the mutant persists in the brain of infected mice for long periods without causing apparent symptoms. Reduced capsule production and growth at 37°C could play a role in making the presence of the fungus in the brain tolerable, as both growth rate and amount of GXM production have been correlated with severity of disease in the mouse (Pool et al., 2013), though data from human patients suggest that the rate of uptake by macrophages is more important than capsule production (Sabiiti et al., 2014). The mutant has normal melanin production, which suggests its entrance into the central nervous system would not be impaired. If it does not cause significant damage and does not trigger a strong inflammatory response, its clearance may be slower as a consequence.

As for transcriptional profiling, while we found few differentially expressed genes in comparison with the subsets reported for Ryp1 and Wor1 in *H. capsulatum* and *C. albicans* (Tuch et al., 2010; Beyhan et al., 2013), this may be due to our choice of an early time point; it is possible that more genes would show changes in their transcript levels in the mutant strain at later times. We also note that most of the genes were downregulated in the mutant, and relatively few were upregulated. Because earlier transcript changes tend to be caused by direct action of the transcription factor under study, and later changes, by indirect action, such as that of TFs regulated by the first one (Haynes et al., 2013), our data strongly suggest that Rcv1 is primarily a transcriptional inducer rather than a repressor.

The largest changes in transcript levels were found in genes of the *MAT*α locus, the majority of which were almost or completely repressed in the absence of Rcv1. Intriguingly, there was close correspondence between the genes that were repressed in the *rcv1*Δ and the ones that are transcriptionally induced when *C. neoformans* is taken up by macrophages (Fan et al., 2005). These include genes that have been correlated with the observed phenotypes of the *rcv1*Δ strain, as shown in Table 2. The *ste20*α mutant has a defect in cytokinesis at 39°C, which occurs in the *rcv1*Δ mutant at 37°C, a temperature at which *ste20*α has a normal phenotype (Wang et al., 2002). Like *rcv1*Δ, *ste20*α has a virulence defect, although it is less pronounced. The *ste12*α mutant retains virulence in a serotype A background, but grows a thinner capsule than the wild-type strain (Yue et al., 1999). In a serotype D background, the *ste12*α mutant is hypovirulent and hypocapsular, but in contrast with *rcv1*Δ, it shows a lower expression level of the laccase 1 gene and lower phospholipase activity on egg yolk agar (Chang et al., 2000). As shown above, *rcv1*Δ also has a capsule defect. The *rum1*α mutant shows reduced lung colonization in the mouse and is hypovirulent to *Galleria* (Jung et al., 2015). However, the deletion of three of the *MAT*α locus genes–*SXI*α (Hull et al., 2002), *STE11*α (Clarke et al., 2001), *STE20*α (Wang et al., 2002)–results in sterile phenotypes that fail to initiate mating (as assessed by filamentation). Unexpectedly, *rcv1*Δ mating partners filament normally as shown in Figure S8, and generate viable spores. Because we have not performed meiosis assays, this observation can mean that mating or unisexual sporulation is occurring in the mutants. However, mutants of genes controlled by Rcv1 in non-mating conditions, such as those of *STE12*α (Yue et al., 1999) and *STE11*α (Davidson et al., 2003) are also incapable of unisexual filamentation. At present, we do not know the mechanism whereby Rcv1 regulates expression of these genes in non-mating conditions.

**Table 2:** Correspondence between observed phenotypes of the *rcv1*Δ strain and those of other *MAT*α locus gene mutants.

***rcv1Δ* phenotype**	**Known *MAT locus* gene mutant with the same phenotype**	**References**
Capsule growth deficit	*ste12α*	Yue et al., 1999
Cytokinesis defect	*ste20α* (39°C)	Wang et al., 2002
Virulence defect (mouse)	*ste20α*	Wang et al., 2002
	*ste11α*	Clarke et al., 2001
Defect in lung colonization	*rum1α*	Jung et al., 2015

The correspondence of 15 genes in two very different experimental setups indicates that at least this part of the *MAT*α locus is co-regulated in processes other than mating. It was previously postulated that the *MAT* locus may represent the convergence of the virulence and mating pathways (Fan et al., 2005). The environmental cues that trigger virulence phenotypes would overlap, to a certain extent, with the ones that trigger mating, and the *MAT* locus would be at the center of both regulatory networks (Nielsen and Heitman, 2007). Fungi mate and sporulate in response to environmental stresses and mating itself is evolutionarily understood as a process that increases genetic diversity and thus, the chances an organism has of acquiring traits necessary to overcome a challenge (Nielsen and Heitman, 2007). It may also serve the purpose of generating quiescent and dispersion forms (spores) that may ensure survival of the organism or allow it to move to another place where conditions are more favorable to growth. It has been suggested that the interior of the macrophage, being widely understood as a nutritionally poor environment, would trigger adaptive processes in the fungus that go through the *MAT* locus but not by initiating cell fusion and mating (Fan et al., 2005).

The data from both the macrophage study and the present one suggest that the *MAT*α locus is a gene cluster that responds both to mating and stress (Fan et al., 2005). We hypothesize that Rcv1 is the switch of the stress branch: it does not respond to mating signaling, but keeps the basal expression level of the portion of the *MAT*α locus that is needed for the responses to heat, starvation and possibly other stress agents. By this hypothesis, the virulence roles of the Ste pathway, and of the *MAT*α locus as a whole, are under the control of a mating-unrelated signaling pathway that works through Rcv1 in *C. neoformans*.

In conclusion, the present work has identified a role of the Rcv1 protein in virulence and adaptation to the host. We have found Rcv1 has a putative DNA-binding domain, localizes to the nucleus and influences the transcriptome of the fungus. Ongoing ChIP-Seq studies using Rcv1 are being pursued to identify its binding site. Given the role we have shown of Rcv1 as a regulator of virulence, identifying the pathway that regulates this transcription factor becomes of paramount importance, not only because some of the proteins involved could possibly be used as drug targets for antifungal therapy, but because it could shed light on the evolution of sex related genes as a mechanism to cope with adversity. Similarly, the regulatory targets of Rcv1 can prove equally promising as therapeutic targets. Our group is currently addressing these issues. We believe Rcv1 opens new avenues of research on the mechanisms of adaptation of *C. neoformans* to the mammalian host.

## Methods

### Strains

The wild-type strains used were H99, KN99a, and KN99α. Deletion mutants for the *RCV1* gene were generated from each (see below). The H99 *rcv1*Δ derivative was used for reconstitution and generation of the strain for cytolocalisation (see below). All strains were kept on solid YPD agar plates at 30°C prior to experiments. In all cases, yeast cells came from an overnight, single-colony, 5 mL of YPD culture made at 30°C and 250 rpm.

### Generation of *rcv1Δ* mutants and reconstitution of the *RCV1* gene

A deletion construct containing the *HPH* Hygromycin B resistance gene cassette flanked by 5′ and 3′ regions of the *RCV1* locus was generated by overlap PCR (Davidson et al., 2002). Primers used in this work are listed in **Table S1**. All PCRs were performed using the Phusion Hot Start II DNA Polymerase (Thermo Fisher Scientific) following supplier's instructions. The *HPH* cassette used as template came from the pPZP-Hyg2 vector (Walton et al., 2005). The deletion cassette was transformed into H99 and KN99 (a and α strains) by biolistics (Toffaletti et al., 1993). Mutants were selected on YPD agar plates containing hygromycin B at 200 μg/mL.

For reconstitution of the wild-type genotype, we generated by PCR an amplicon containing the intact *RCV1* locus using the outermost primers of the deletion cassette and H99 genomic DNA as template. That amplicon was transformed by biolistics into the *rcv1*Δ mutant strain alongside the pJAF1 plasmid containing the Neo^r^ neomycin resistance cassette (Fraser et al., 2003). Reconstitution candidate colonies were selected for on YPD agar plates containing G418 (Sigma-Aldrich) at 200 μg/mL.

Mutants were confirmed by Southern blotting and most confirmations were performed using the DIG System (Roche Applied Science): PCR DIG Labeling Mix for generating the probe, DIG Wash and Block Buffer Set for processing the blotting membrane and CDP-Star, ready to use, for generating the signal. However, the *rcv1*α mutant was confirmed using the AlkPhos^®;^ System (GE Life Sciences). The membrane itself was of positively charged nylon (HyBond N+, GE Life Sciences) and detection of the signal was by an X-Ray film (Hyperfilm ECL, GE Life Sciences). The enzymes used to digest genomic DNA were NcoI and PvuII (New England Biolabs) and the genomic DNA was extracted by the lyophilisation method (Pitkin et al., 1996).

The *rcv1* + *RCV1* strain was validated by real-time PCR to confirm the expression of the *RCV1* gene. The condition chosen for RNA extraction was vegetative growth on YPD at 30°C. For details of the procedure, see the real-time validation section below.

### Phenotype assays

The *rcv1*Δ mutant was tested for several stressors and virulence factors *in vitro*. For serial dilution assays, we used five-spot series at 10-fold dilutions from one spot to the next, starting at 10^5^ cells in the first spot. For single-spot assays, we used 10^5^ cells. Assays were carried out at 30°C unless stated otherwise. Cells were washed once by centrifugation (1,000 *g*, 5 min, room temperature) and resuspension in deionised, sterile water, and cell density was calculated by haemocytometer counting.

To assess melanisation, a single-spot assay was carried out on L-DOPA agar (29.4 mM KH_2_PO_4_, 10 mM MgSO_4_, 13 mM glycine, 15 mM dextrose, 3 μM thiamine, 2 mM L-DOPA, 1.5% agar) for 72 h. For urease production, a single-spot assay was carried out on urea agar (0.2% peptone, 86 mM NaCl, 14.7 mM KH_2_PO_4_, 333 mM urea, 45 μM Phenol Red, pH 6.9) for 48 h. For phospholipase secretion, a single spot assay was carried out on egg yolk agar (8% egg yolk, 0.8% peptone, 1.6% dextrose, 0.84 M NaCl, 42 mM CaCl_2_, 1.6% agar) for 48 h. For urease and phospholipase, the presence of halos around the spot colony was assessed: a pink halo on urease plates, and a translucent halo on phospholipase plates.

To assess resistance to stressing agents, we used serial dilution spots: for salt stress, YPD with NaCl or KCl at 1 M; for cell wall stress (30 and 37°C), with Congo Red at 1%; for oxidative stress, with menadione at 1 mM.

Capsule formation was assessed on DMEM-MOPS agar at 37°C and 5% CO_2_, which was also used to assess viability of the mutant on a serial dilution assay (Gilbert et al., 2010). We also used Sabouraud/MOPS in capsule studies (Zaragoza and Casadevall, 2004). Cells were resuspended in 50% suspension of Percoll^®;^ in water (Paes et al., 2018) and inspected under DIC in a Zeiss Z1 Axio Observer inverted microscope using a 40X objective (EC Plan Neofluar 40X/0.75 Ph 2; Carl Zeiss GmbH) and an MRm cooled CCD camera (Carl Zeiss GmbH). Images were collected with the Zen 2012 software. We measured diameter of capsule and cell of 50 yeasts of each strain and compared the mean volume ratios of capsule and cell among them.

### Cell morphology analyses

Macroscopic and microscopic cell morphology was observed in two capsule-inducing media: CO_2_-independent medium [CIM; (Ost et al., 2015)] and 10-fold diluted Sabouraud in 50 mM MOPS [Sab/MOPS; (Zaragoza and Casadevall, 2004)]. CIM was also used to document cell division by bright field, time-lapse microscopy. To do that, cells were seeded onto a well of a 96-well plate, at a density of a thousand cells per well in 100 μL of CIM. Pictures were taken every 30 min in the same microscope as above, over a course of 48 h at 37°C using a 10X objective. A sample of each culture was also applied to a microscope slide for inspection at 63X (Plan-Apochromatic 63X/1.40 DIC; Carl Zeiss GmbH) using differential interference contrast (DIC). Images were captured with an Mrm CCD camera (Carl Zeiss GmbH) and the whole microscope and camera were operated for image capture by the Zen 2012 software.

Cells from these cultures were also sonicated using a QSonica Q700 Sonicator equipped with a 1.6 mm tip. The settings were 10% amplitude for intervals of up to 1 min, when all cells were destroyed. At intermediate times, aliquots were inspected under bright field microscopy to assess whether cell clumps had dispersed.

### Growth rate measurement

Yeast cells, previously washed with deionised water to remove culture medium and counted, were diluted in YPD or CIM at a concentration of 10^4^ cells/mL and 100 μL of each suspension were seeded in triplicate onto flat-bottom 96-well plates, including medium itself as a negative control. Plates were then incubated at 30 or 37°C in an EON Microplate Spectrophotometer (Biotek Inc) set to double-orbital shaking at 500 rpm, and the optical density at 600 nm was measured every 30 min. Measurements for each strain and time point were averaged and plotted.

### Cytolocalisation of the Rcv1 protein

We constructed a *RCV1*-GFP chimera by cloning the *RCV1* coding sequence into the pCN50 plasmid, which contains the *NAT* nourseothricin resistance marker (O'Meara et al., 2010). To do that, total RNA from the H99 strain (grown overnight on 5 mL of liquid YPD at 30°C) was extracted using TRIzol^®;^ (Thermo Fisher Scientific). Briefly, cells were pelleted by centrifugation (1,000 *g*, 5 min, 4°C), washed with deionised water and recentrifuged, resuspended in 500 μL of TRIzol^®;^, 500 μL of glass beads (300 μm) were added and the suspension was vortexed at maximal speed for 15 min. From there the protocol proceeded as outlined by the supplier, using BCP (Sigma-Aldrich) to generate the aqueous phase.

Reverse transcription was performed using the High Capacity cDNA Synthesis Kit (Thermo Fisher Scientific) on 2 μg of total RNA, followed by PCR-amplification of the coding region for the *RCV1* gene. Two 15-base tails were added to the primers for directional cloning into the SpeI site of pCN50 (the restriction enzyme came from New England Biolabs) by recombination using the In-Fusion HD EcoDry Cloning Plus Kit (TaKaRa Clontech) according to supplier's instructions. The recombination reaction was transformed by heat shock into HST08 *E. coli* competent cells (TaKaRa Clontech) and the recombinant plasmid was extracted from resistant colonies and transformed into the *rcv1*Δ mutant strain. The resulting strain, *rcv1* + *RCV1-GFP*, was obtained by selecting transformants for their resistance to nourseouthricin (Jena Bioscience; 200 μg/mL) and screening them microscopically for green fluorescence using the parental strain as negative control. For nuclear staining, cells were incubated for 30 min at 37°C with the cell-permeant DNA stain Hoechst 33342 (NucBlue^®;^ Live Reagent, Thermo Fisher Scientific) prior to microscopy. We used a 63X objective with immersion oil and the 38 HE filter for GFP (excitation 470/40 nm, emission 525/50 nm) and 49 DAPI for Hoechst (excitation maximum 365 nm, emission 445/50 nm; Carl Zeiss GmbH).

### Cryptococcus killing assay

The mutant was tested for its ability to proliferate within macrophages relative to the wild-type and reconstituted strains. We used J774A.1 (originally purchased from the American Type Culture Collection, ATCC^®;^ code TIB-67™) cells without previous stimulation, and the procedure was as previously (Nicola and Casadevall, 2012) outlined, with the following modifications: (a) the MOI was of five yeast cells per macrophage; and (b) after 2 h of phagocytosis, unphagocytosed cells were removed by a washing step with 1X PBS. The opsonin used was the 18B7 monoclonal anti-GXM IgG (Zebedee et al., 1994; kindly supplied by Arturo Casadevall) at 10 μg/ml. The phagocytic index was defined as the percentage of macrophages that have taken up yeast cells in the 2 h of incubation prior to washing. The experiment was independently performed three times.

*Acanthamoeba castellanii* strain 30234 was cultured according to Moffat and Tompkins (1992). For the cryptococcal phagocytosis and killing assay, 5 × 10^4^ amoebae were exposed to yeast cells at a MOI of two in 200 μL of PYG medium. Unphagocytosed cells were washed off after 2 h. After 24 h at 30°C, the supernatants were transferred to microfuge tubes and amoebae were lysed for 10 min in 50 μL of a 0.05% deoxycholate solution, after which the lysate was added to the collected supernatants and serial dilutions (10^−1^ to 10^−3^) were plated on YPD agar for colony counting. The experiment was performed in triplicate.

### Detection of surface GXM

Cells grown for 24 h in Sab/MOPS at 37°C were treated with the 2D10 monoclonal, anti-GXM IgM (Zebedee et al., 1994; kindly supplied by Arturo Casadevall): 10^6^ cells, antibody at 10 μg/mL, 30 min at 4°C, then washed with PBS prior to adding the secondary Ab, Alexa Fluor^®;^ 488-conjugated polyclonal goat IgG against mouse IgM (Thermo Fisher Scientific) at 1 μg/mL for 30 min at 4°C and washing with PBS. The cells were then applied to a glass slide and documented under the microscope described above. We used a 63X objective with immersion oil and the 38 HE excitation/emission filter (Carl Zeiss GmbH).

### ELISA quantitation of secreted GXM

The strains were seeded at a concentration of 10^6^ cells/mL on 50 mL minimal medium (29.4 mM KH_2_PO_4_, 10 mM MgSO_4_, 13 mM glycine, 3 μM thiamine, 15 mM dextrose) and incubated at 37°C at 150 rpm. Samples were collected daily for 5 days and processed for GXM dosing by ELISA as described previously (García-Rivera et al., 2004).

### *In vivo* virulence assays

To test the mutant for its ability to cause disease, we used both the murine and *Galleria mellonella* (Mylonakis et al., 2005) models. For mice, a suspension of 2 × 10^5^ cells in ten microliters of PBS was administered intratracheally to each anesthetized animal (9–12 BALB/c individuals per group, all at 8 weeks of age at the time of administration; the anesthetic was a ketamine/xylazine mixture administered intraperitoneally). Animals were then weighed daily and scored for signals of illness. The criteria were weight loss as a percentage of the original weight (10–20%, one point; 20–25%, two points; >25%, three points), decrease in spontaneous (one point) and stimulated (two points) activity, ruffled hair coat (one point) and the presence of neurological symptoms (macrocephaly, two points; rear limb paralysis, three points). When animals reached a score of four, they were euthanised by CO_2_ asphyxiation.

For *Galleria*, last instar larvae in the 200 mg weight range were injected in the terminal left proleg with 10^4^ yeast cells in ten microliters of PBS containing ampicillin at 400 μg/mL. Sixteen to twenty individuals were infected per group. Two experiments were performed differing only in the temperature the caterpillars were kept after infection, 30 or 37°C. Deaths were counted daily and the experiment was terminated when all individuals in the PBS group molted. Molted individuals in any group were censored from the analysis at the day of their molting.

### Ethics statement

All mouse experiments were pre-approved by the Committees for Use of Animals in Research of the Catholic University of Brasília (protocol 018/14) and of the University of Brasília (Doc. No. 52657/2011), in conformity with the guidelines of the Brazilian National Council for Control of Animal Experimentation (CONCEA).

### Brain and lung fungal burden

Five-animal mouse groups were infected as described above and sacrificed at 12 days of infection by CO_2_ asphyxiation. Brain and lungs were removed surgically. The brain and the right lung were weighed and then macerated in PBS separately and a three-step 10-fold dilution series was prepared for each organ. Each dilution was plated in triplicate onto YPD agar plates and these were incubated for 48–72 h at 30°C. Colonies were counted and the mean of the replicates for each individual were used to calculate the fungal burden in each organ. The same assay was performed at later time points for the mutant strain (because the wild-type and reconstituted strains cause the animals to die much earlier).

### Histopathology

To compare the tissue damage in the lung caused by the mutant strain to the wild-type one, we used the left lungs of the sacrificed animals from the fungal burden assay. Using a 1-milliliter syringe, they were inflated with a 1% paraformaldehyde solution in PBS and then immersed in the same solution for complete fixation. Later the lungs were embedded in paraffin and tissue sections were mounted on glass slides and processed for microscopy with haematoxylin and eosin. Images were collected with an MRc5 color CCD camera (Carl Zeiss GmbH) on the microscope described above.

### RNA-seq and real-time PCR validation

Because most of the phenotypes observed for the mutant appeared when it was incubated in CIM at 37°C, we chose these incubation conditions at an early time point of 4 h to compare the wild-type and mutant strains for their gene expression profiles. Four separate samples of the wild-type and two of the mutant were generated: for each, a 50-mL pre-warmed CIM sample was inoculated with yeast cells at a final concentration of 9 × 10^6^ cells/mL and incubated for the aforementioned periods under shaking at 150 rpm. Then the cultures were rapidly cooled in an ice-water bath, cells were pelleted by centrifugation (1,000 *g*, 4°C), the supernatant was poured out and the pellet was snap-frozen in an ethanol-dry ice bath. Pellets were cryo-desiccated and total RNA was extracted using the RNEasy^®;^ Plant RNA Extraction Kit (QIAgen) with a slightly modified version of the standard protocol, namely that 500 μL of glass beads (0.6 cm) were added to the cryodesiccated pellet, which was then vortexed at maximal speed until it was a fine powder. At that point, 600 μL of the RLT buffer of the kit were added to the powder and from there the extraction proceeded as directed by the supplier.

Total RNA was sent to the Duke University Center for Genomic and Computational Biology, where it was submitted to poly-A+ RNA purification (KAPA Stranded mRNA-Seq Kit, Kapa Biosystems) and sequenced using the Illumina dye sequencing method in a HiSeq 2000 machine (Illumina, Inc). The sequencing protocol was single-end 50 bp. Raw sequencing data were assembled using the Tophat 2 software (Kim et al., 2013) and transcript quantitation was calculated via the Cufflinks/Cuffdiff pipeline (Trapnell et al., 2012) using default parameters. The reference genome and transcriptome of the wild-type strain were retrieved from database of the *C. neoformans* H99 Sequencing Project at the Broad Institute of MIT and Harvard. Gene ontology analysis was performed with the Blast2GO software (Conesa et al., 2005). The FASTQ files containing sequence data were deposited at the Sequence Read Archive (http://www.ncbi.nlm.nih.gov/Traces/sra/) under accession number SRP067741.

Genes of interest that were shown to be differentially expressed by RNA-Seq were submitted to validation by real-time PCR using both the original samples and two RNA samples extracted *de novo*. Total RNA was reverse transcribed (2 μg per sample) using the High Capacity cDNA Reverse Transcription Kit following supplier's instructions. The real-time PCR itself was carried out in a 7500 Fast Real-time PCR System using the Fast SYBR Green Master Mix. All reagents and the instrument were supplied by Thermo Fisher Scientific. Forward primers of all targets spanned an exon-exon junction and this allowed us to circumvent DNAse treatment. The housekeeping normaliser was the *ACT1* gene that codes for the alfa-subunit of actin, and the reference condition was H99 at the same time point. Changes in the relative transcript quantity were calculated by the ΔΔCt method.

### Mating assay

Strains were co-suspended in water at a concentration of 10^7^ yeast cells/mL for each mating partner, and 10 μL were applied to a spot on a SLAD agar plate (0.17% yeast nitrogen base without amino acids, 50 μM (NH_4_)_2_SO_4_, 20 g/L dextrose, 20 g/L agar). The spots were made in triplicate for each mating pair. The plates were kept in the dark at room temperature and filamentation was documented daily by photographing the whole spot on a stereomicroscope and the filamentation sites, under a 10X objective in an upright microscope. The same experiment was performed independently in Murashige & Skoog agar (MSA), and basidiospores were collected using a yeast tetrad dissection microscope and seeded onto an YPD agar plate to assess viability by germination.

## Author contributions

HP designed and performed experiments, analyzed data and wrote the paper. LD participated in the mouse experiments. LP assisted in generation of the KN99 mutants. PA participated in the *Galleria* and *Acanthamoeba* experiments and performed measurements of secreted GXM. AN performed part of the microscopy experiments. MV assisted in initial gene disruption experiments. GP performed gene ontology classification of differential genes. FS helped validate RNA-Seq data by PCR. JA assisted in RNA-Seq design and execution, and performed the MSA mating. MF supplied funding, assisted in data analysis and oversaw writing of the paper. LF conceived the project, generated the KN99 mutants, performed initial phenotype analysis and analyzed data.

### Conflict of interest statement

The authors declare that the research was conducted in the absence of any commercial or financial relationships that could be construed as a potential conflict of interest.
