# Supplementary figures and images for: A Wor1-Like Transcription Factor Is Essential for Virulence of Cryptococcus neoformans
Source: Front Cell Infect Microbiol. 2018 Nov 13;8:369. doi: 10.3389/fcimb.2018.00369 (PMC6243373; doi:10.3389/fcimb.2018.00369)

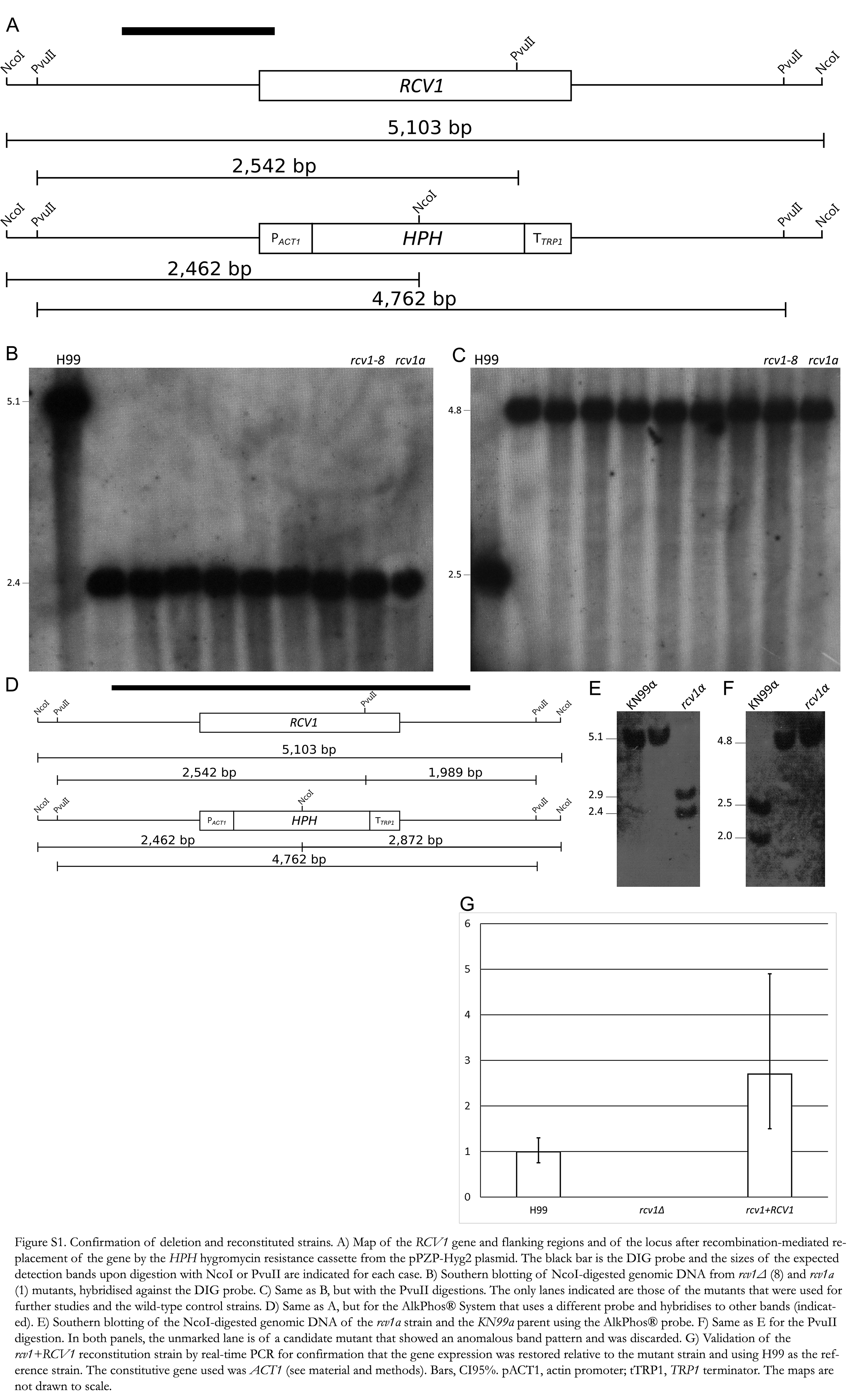

Supplement: Supplementary file 1 [file Image_1.tif]

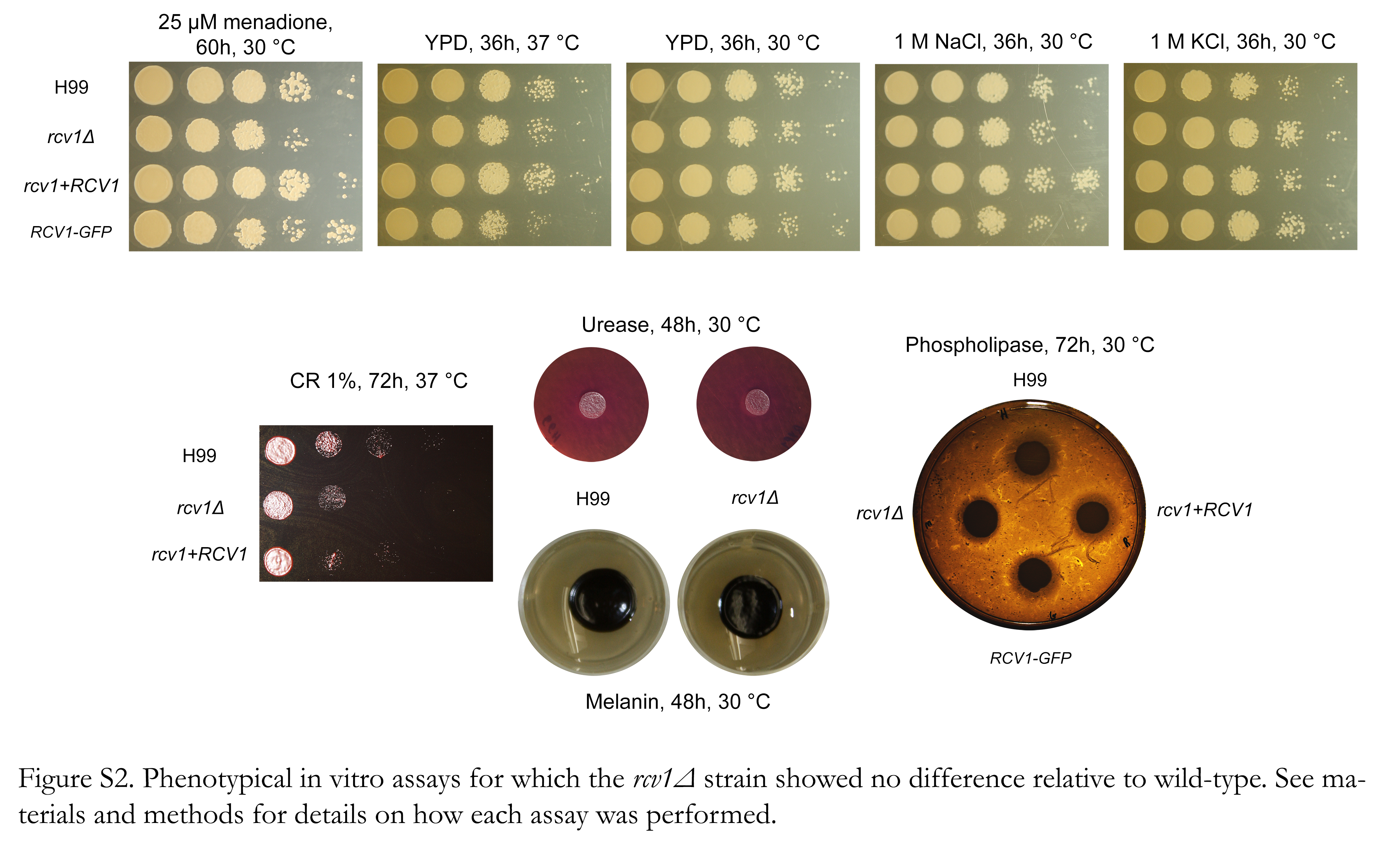

Supplement: Supplementary file 2 [file Image_2.TIF]

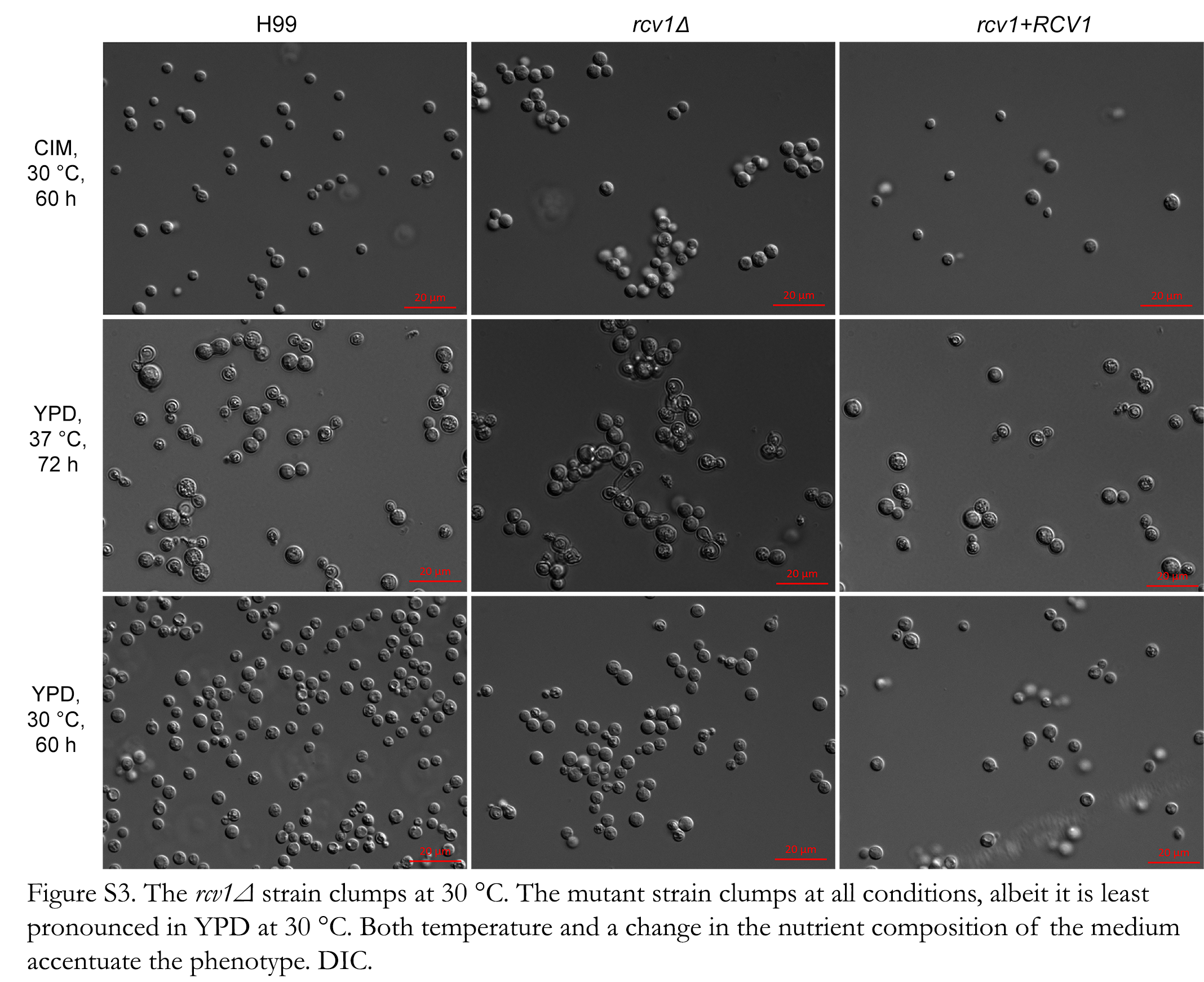

Supplement: Supplementary file 3 [file Image_3.TIF]

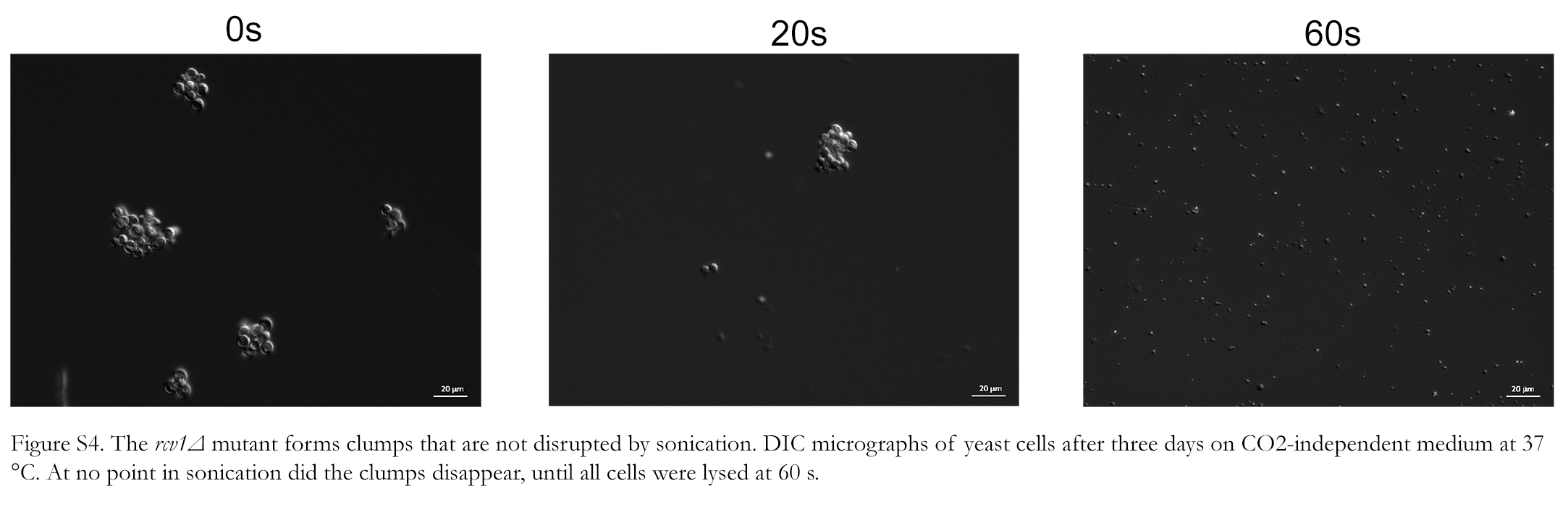

Supplement: Supplementary file 4 [file Image_4.tif]

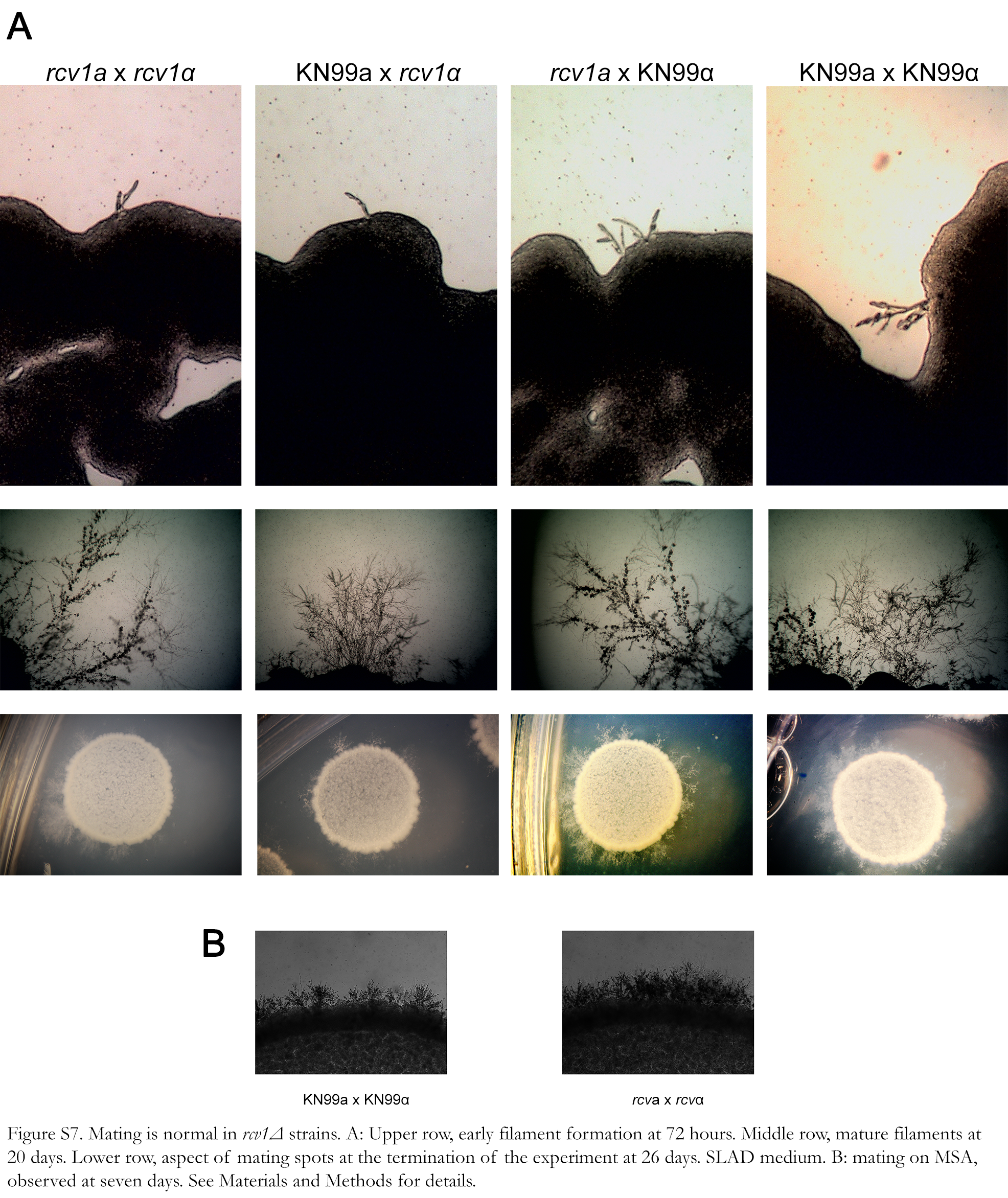

Supplement: Supplementary file 7 [file Image_7.TIF]

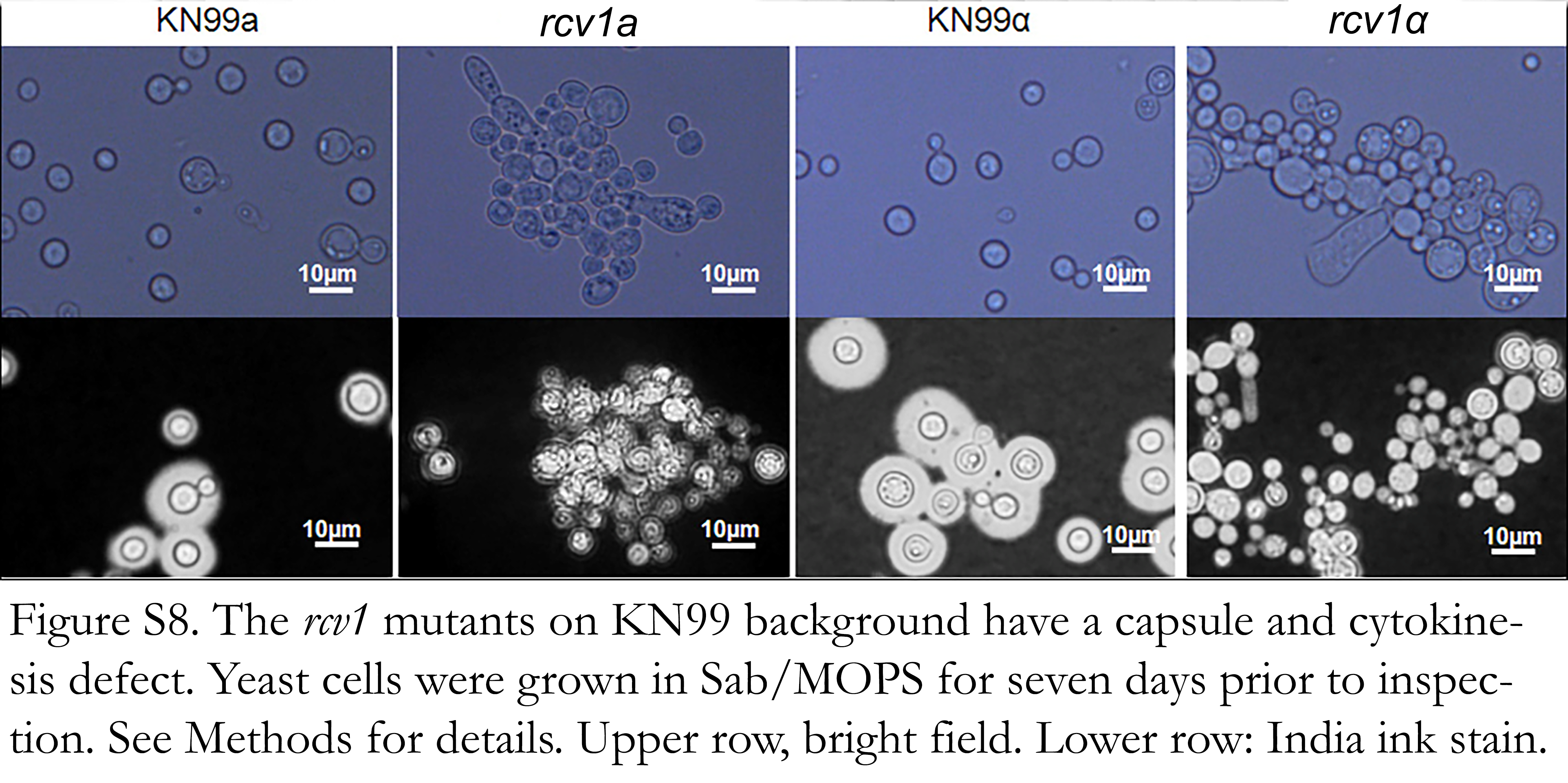

Supplement: Supplementary file 8 [file Image_8.TIF]
