# Supplementary material for: A Wor1-Like Transcription Factor Is Essential for Virulence of Cryptococcus neoformans
Source: Front Cell Infect Microbiol. 2018 Nov 13;8:369. doi: 10.3389/fcimb.2018.00369 (PMC6243373; doi:10.3389/fcimb.2018.00369)
Supplement: Supplementary file 5 [file Image_5.pdf]

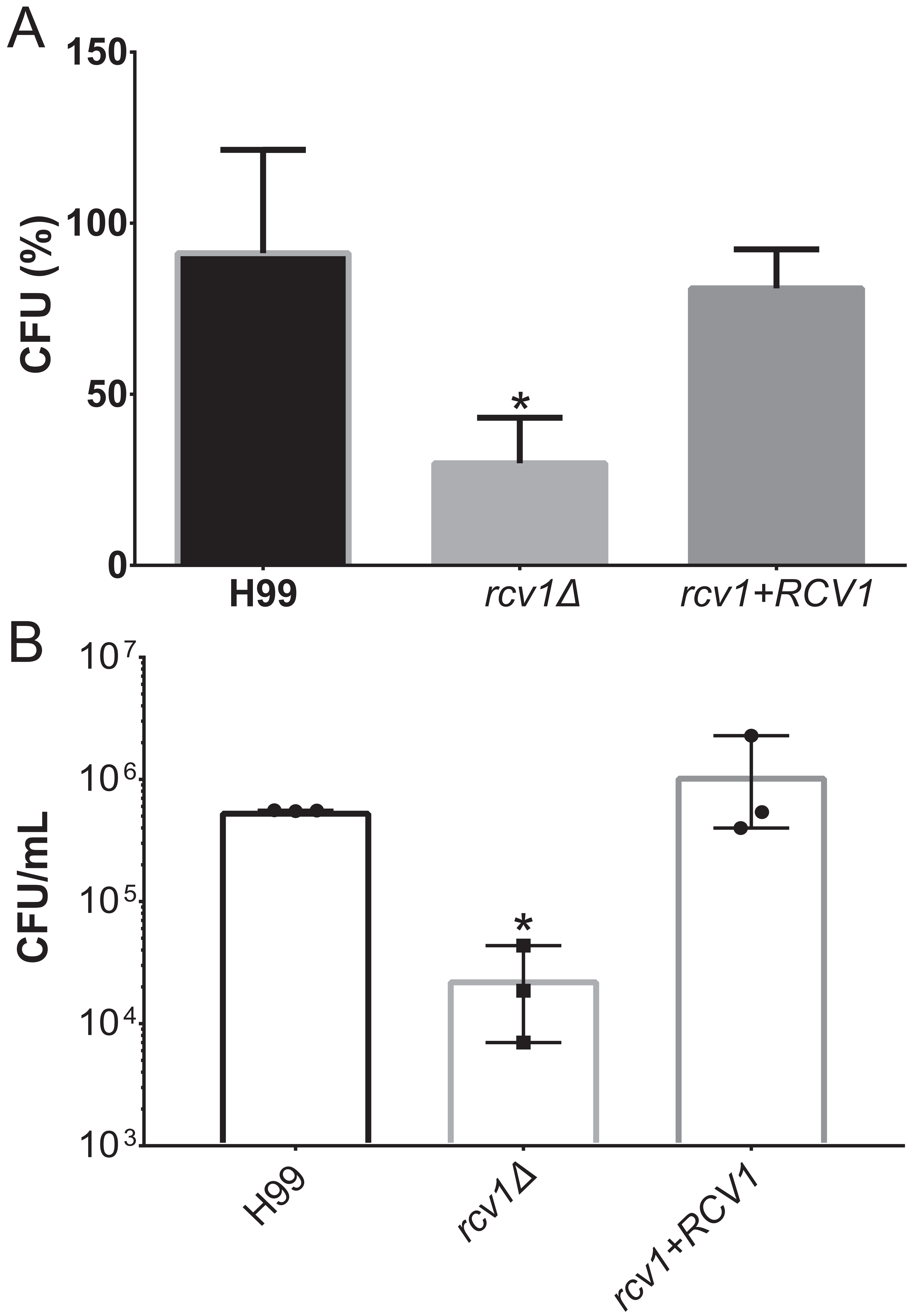

Figure S5. The *rcv1Δ* strain shows reduced CFU numbers after incubation with J774A.1 macrophages or amoebae than the parental strain. (A) Yeast cells were opsonised with 18B7 antibody and added in triplicate at a multiplicity of infection (MOI) of five to macrophage monolayers and colony-forming unit (CFU) plates were made the next day. The CFUs were counted from 100 µl aliquots of 1,000-fold dilutions of cell lysates, and the mean values are plotted in histograms as percentages of the mean value for the H99 strain, which was set at 100%. Bars, CI95%. The asterisk indicates statistical significance relative to the other two strains (one-way ANOVA, Tukey's post-test, $p<0.0001$). This experiment is representative of three. (B) Yeast cells were incubated with *A. castellanii* strain 30234 amoebae at a MOI of two and after one day at 30 °C, CFU plates were generated and counted as for macrophages. Three replicates are represented as dots for each co-incubation. Columns are means and bars are ranges. Because of an outlier on the reconstituted strain, ANOVA yielded negative results, but the asterisk indicates a statistically significant difference ($p = 0.0002$) for a two-tailed Student's t-test with Welch's correction comparing the mutant and wild-type strains.
