# Supplementary material for: A Wor1-Like Transcription Factor Is Essential for Virulence of Cryptococcus neoformans
Source: Front Cell Infect Microbiol. 2018 Nov 13;8:369. doi: 10.3389/fcimb.2018.00369 (PMC6243373; doi:10.3389/fcimb.2018.00369)
Supplement: Supplementary file 6 [file Image_6.pdf]

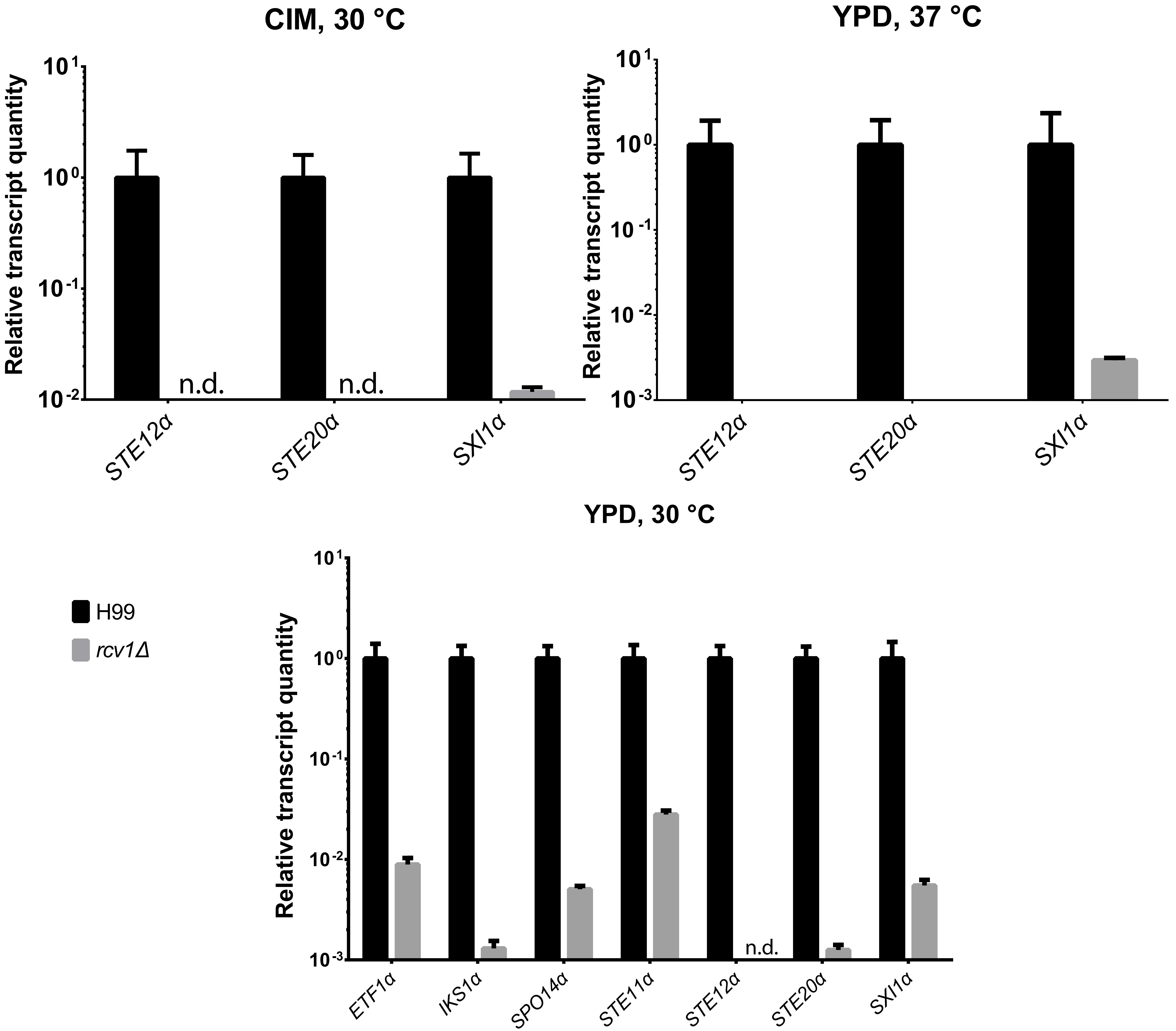

Figure S6. Quantitative PCR shows that Rcv1 regulates *MATa* locus genes under vegetative growth and different incubation conditions. The plots indicate relative transcript quantitation of the indicated ORFs, using *ACT1* as the housekeeping transcript and, for each incubation condition, the H99 samples as reference. Bars are SDs. The experiments were performed twice. N.d., not detectable (transcripts were less than 0.01% of wild-type). The y axis is in a log-10 scale.
