# Supplementary material for: A Wor1-Like Transcription Factor Is Essential for Virulence of Cryptococcus neoformans
Source: Front Cell Infect Microbiol. 2018 Nov 13;8:369. doi: 10.3389/fcimb.2018.00369 (PMC6243373; doi:10.3389/fcimb.2018.00369)
Supplement: Supplementary file 9 [file Image_9.pdf]

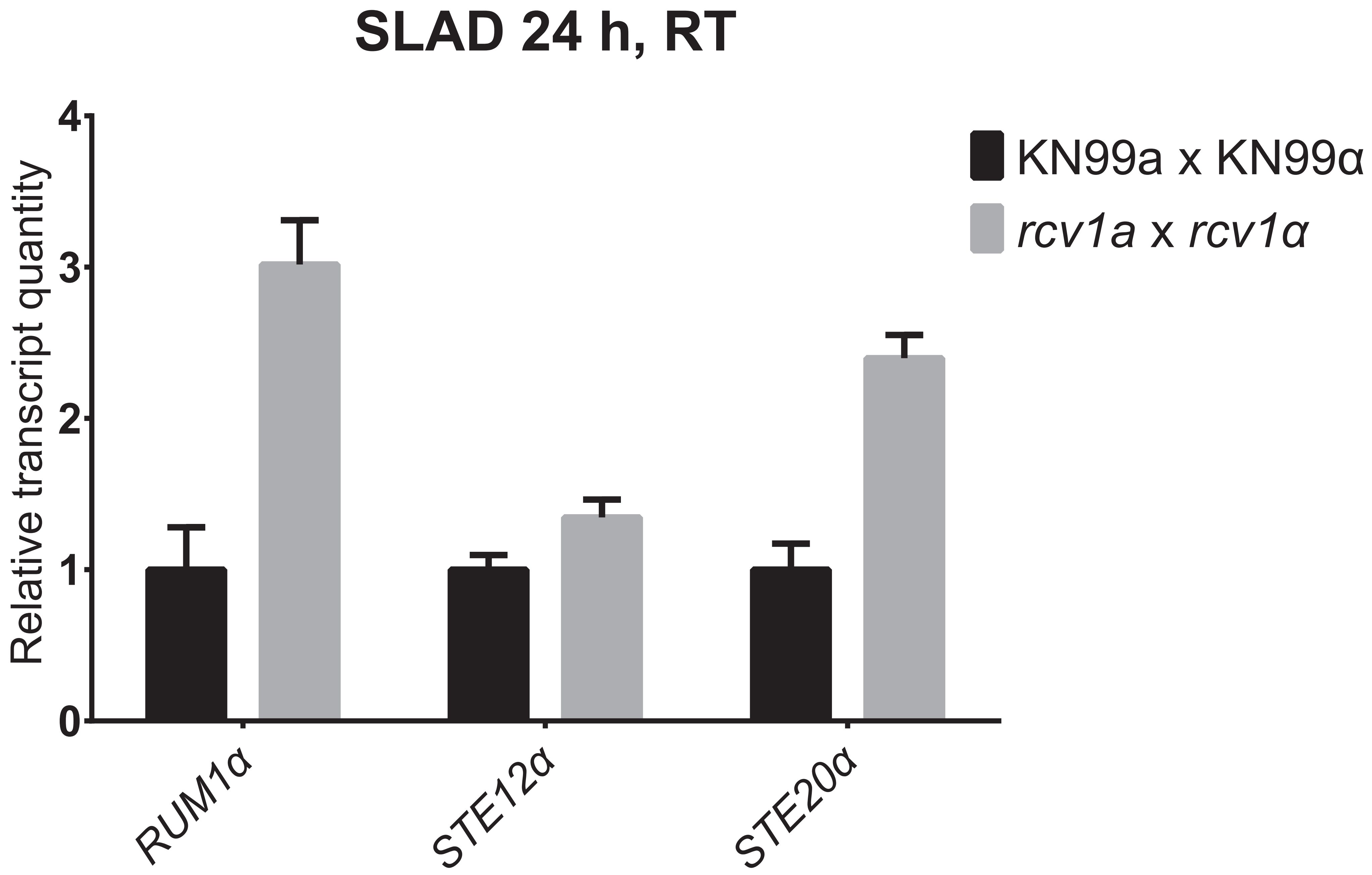

Figure S9. The *MATa* locus genes linked to the *rcv1Δ* phenotypes are not repressed in the mutant under mating conditions. The plots indicate relative transcript quantitation using *ACT1* as the housekeeping transcript and the wild-type mating condition as reference. Bars are SDs.
